# Vitamin D promotes apoptosis and enhances cisplatin sensitivity in bladder cancer cells by inhibiting the Warburg effect through the AKT/mTOR pathway

**DOI:** 10.1186/s12894-025-01994-2

**Published:** 2025-12-15

**Authors:** Jian Zhou, Chaoyang Zhang, Xiao Wang, Ji Xing, Geng Cheng, Hao Chu

**Affiliations:** 1 Department of Urology, Wuhan Third Hospital (Tongren Hospital of Wuhan University), Wuhan, China; 2 Department of Urology, Wuhan University Affiliated Tongren Hospital, Wuhan, China

**Keywords:** Bladder cancer, Vitamin d, Cisplatin, Warburg effect, AKT/mTOR pathway

## Abstract

**Objective:**

Patients with bladder cancer (BCa) have a poor prognosis and are prone to metastasis. Deficiency of 1,25-dihydroxyvitamin D3 (VD) is associated with increased incidence and decreased survival in various tumors. Herein, we aimed to examine the effect of VD combined with cisplatin (DDP) on the proliferation and apoptosis of BCa cells and elucidate the underlying mechanism.

**Methods:**

T24 and 5637 BCa cell lines were treated with different concentrations of DDP and VD to assess the effects of various doses of DDP and VD on BCa cytotoxicity and determine the appropriate combination dose. T24 cells were treated with DDP and VD to assess the effects of the drug combination on cell proliferation, apoptosis, cycling, Warburg effect, and DDP sensitivity. In addition, cells were treated with DDP, VD, pyruvic acid sodium (PAS), or SC79 to determine the effect of VD on the sensitivity of BCa cells to DDP mediated by inhibiting the Warburg effect through AKT/mTOR signaling.

**Results:**

VD and DDP inhibited BCa cell proliferation; promoted apoptosis; downregulated the protein expression of GLUT1, LDHA, HK2, c-Myc, MRP1, and P-gp; and upregulated the expression of p-mTOR protein. VD combined with DDP reversed the effects of PAS on cells and promoted apoptosis by inhibiting the cellular Warburg effect. In addition, VD combined with DDP activated the AKT/mTOR pathway and reversed the effects of SC79 on cell proliferation and the Warburg effect.

**Conclusion:**

VD could promote apoptosis and enhance DDP sensitivity in BCa cells by inhibiting the Warburg effect via the AKT/mTOR pathway.

**Supplementary Information:**

The online version contains supplementary material available at 10.1186/s12894-025-01994-2.

## Introduction

Bladder cancer (BCa) is the most common malignant tumor of the urinary tract and one of the ten most common types of cancer worldwide, accounting for approximately 550,000 new cases annually [1, 2]. BCa is categorized into two types, with approximately 90% deemed non-muscle invasive and a few cases classified as muscle invasive [3]. Cisplatin (DDP)-based chemotherapy is the first-line treatment for most cancers [4]. However, the overall efficacy of DDP remains suboptimal because of complex pathological subtypes, genomic differences, and drug resistance [5–7]. Thus, increasing the efficacy of DDP may improve the overall survival of patients with BCa.

1,25-Dihydroxyvitamin D3 (1,25D3; VD) deficiency is associated with an increased incidence and decreased survival in a variety of tumors, including prostate, ovarian, and oral cancers [8–11]. Low serum levels of VD (circulating VD metabolites) are associated with increased incidence and decreased survival in BCa [12, 13]. VD is an active metabolite of vitamin D that exhibits antitumor properties in numerous cancers and was shown to increase DDP sensitivity in gastric cancer [14]. However, whether VD affects the response of BCa cells to chemotherapy is yet to be established.

The Warburg effect is a well-known feature of cancer-specific metabolism [15]. The development of BCa involves alterations in multiple metabolic pathways, including specific shifts in aerobic glycolysis-dependent metabolism (Warburg), which relies on aerobic glycolysis as the primary energy source to sustain uncontrolled growth and proliferation [16]. The Warburg effect is directly related to the activation of specific oncogenes and the loss of tumor suppressor genes and was found to be crucial for tumor progression and maintenance, as well as for cell transformation [17]. Several proteins involved in glycolysis, including glucose transporter 1 (GLUT1), hexokinase 2 (HK2), and lactate dehydrogenase A (LDHA), are overexpressed in cancers [18]. These enzymes increase glycolysis efficiency. VD reportedly suppresses the development of colon cancer by suppressing the Warburg effect [19]. Myc transcription factors are also regulated by the Raf/Mek/Erk signaling pathway and promote glycolysis [20]. In addition, the AKT/mTOR signaling pathway was found to affect cancer cell metabolism and growth by regulating c-Myc [21]. Nevertheless, whether VD affects BCa progression by regulating the AKT/mTOR signaling pathway remains unknown.

In the present study, we aimed to provide an experimental basis and mechanism through which VD promotes BCa sensitivity to chemotherapy and explore the potential of VD plus DDP combination therapy. Accordingly, we employed BCa cell lines to examine the effect of VD on apoptosis, chemosensitivity, and the targets and pathways associated with the Warburg effect in BCa cells.

## Materials and methods

### Main materials and reagents

VD (HY-10002), DDP (HY-17394), pyruvic acid sodium (PAS; HY-W015913), and SC79 (HY-18749) were purchased from MedChemExpress (New Jersey, US). RPMI-1640 (SH30809.01B) was purchased from HyClone (Logan, Utah, US). Antibodies against Bax, Bcl-2, GLUT1, LDHA, HK2, mTOR, c-Myc, MRP1, P-gp, goat anti-rabbit IgG, and GAPDH were purchased from Bioswamp (Wuhan, China). The anti-p-mTOR antibody was purchased from Abcam (Cambridge, UK). The ATP (A095-1-1) and lactate (A019-2-1) assay kits were purchased from Nanjing Jianchen Bioengineering Institute (Nanjing, China). The glucose (BC2505) was purchased from Solarbio (Beijing, China).

### Cell culture

T24 and 5637 cells were purchased from the Cell Bank of the Chinese Academy of Sciences. The cells were cultured in RPMI 1640 medium supplemented with 10% fetal bovine serum and 1% penicillin-streptomycin at 37℃ under 5% CO_2_.

### CCK-8 assay

Cells were collected from each group, and the cell density was adjusted to 1 × 10^4^ cells/mL. Three replicate wells were established in 96-well plates, and each well was inoculated with 200 µL of cells. Following cell adherence, 100 µL of medium containing 10 µL of CCK-8 was added to the cells, followed by incubation for 2 h. The absorbance of wells was measured at 450 nm using a microplate reader.

### Flow cytometry

Cell cycle: The cells were transferred to a flow-through tube, resuspended in pre-cooled phosphate-buffered saline (PBS), and centrifuged at 1000 rpm for 5 min. Subsequently, pre-cooled 75% ethanol was added to the cells at −20℃ and incubated overnight at 4℃. The cells were rinsed in PBS and centrifuged at 1,000 rpm for 5 min; this was followed by the addition of 0.01% RNase and 0.5% propidium iodide (PI) and incubation at 4℃ for 20 min, and cell-cycle distribution was analyzed by flow cytometry.

Apoptosis: Cells were pipetted into a flow-through tube, resuspended in PBS, and centrifuged at 800 rpm for 5 min. Subsequently, the supernatant was discarded, and the cells were resuspended by adding 250 µL of binding buffer. After adding 10 µL of annexin V-FITC, cells were incubated at room temperature for 15 min and centrifuged at 800 rpm for 5 min; the supernatant was discarded. Next, cells were resuspended in 250 µL of binding buffer, and 0.5% PI (5 µL) was added for apoptosis detection.

### Extracellular acidification rate (ECAR)

The extracellular acidification rate (ECAR) was measured in real-time using the ECAR fluorescence assay kit (E-BC-F069, Elabscience, USA). Cells were seeded into a 96-well black clear-bottom culture plate at a density of 500,000 cells per well, with a working solution volume of 100 µl. After incubation in the dark at 37 °C for 30 min, the samples were analyzed using a fluorescence microplate reader (PerkinElmer/Ensight). The default settings were an excitation wavelength of 490 nm and an emission wavelength of 535 nm, with fluorescence readings taken every 4 min at 37 °C for a total of 100 min.

### Western blot analysis

After collecting cultured cells from each group, proteins were extracted from the TPER lysate, and protein concentrations were determined. The specimens were boiled and denatured for sodium dodecyl sulfate-polyacrylamide gel electrophoresis. The primary antibodies (Bax [1:1000], Bcl-2 [1:1000], GLUT1 [1:1000], LDHA [1:1000], HK2 [1:1000], mTOR [1:1000], p-mTOR [1:1000], c-Myc [1:1000]) were incubated overnight at 4℃. Then, 200 mA was applied for 1 h to electrically transfer the primary antibodies to 0.45 μm PVDF membranes. Subsequently, the membranes were incubated for 1 h with 5% bovine serum albumin, MRP1 [1:1000], P-gp [1:1000], and GAPDH [1:1000] at 4℃ overnight. After rinsing with TBST, a horseradish peroxidase-labeled secondary antibody (goat anti-Rabbit IgG [1:2000]) was added to specimens, followed by incubation for 1 h at room temperature. An ECL chemiluminescent agent was used to develop the color after the sample was rinsed with TBST. LI-COR exposure was used to develop the photographs, and ImageJ software (National Institutes of Health, Bethesda, MD) was used to analyze the grayscale of the protein blot.

### Statistics and analysis

Experiments were conducted with three replicate wells per cell group, and each batch of cells was repeated at least three times. Experimental data were analyzed using the SPSS software (version 17.0; SPSS Inc., Chicago, IL, USA). For normally distributed continuous data, results are presented as the mean ± standard deviation. For comparisons among multiple groups, one-way ANOVA was used, and pairwise comparisons were performed using the t-test. The chi-square (χ²) test was used to analyze categorical data. Graphs were generated using GraphPad software (version 8.0; GraphPad Software, Inc., La Jolla, CA, USA). A P-value of < 0.05 was considered statistically significant.

## Results

### Screening of VD and DDP concentrations

**Fig. 1:**
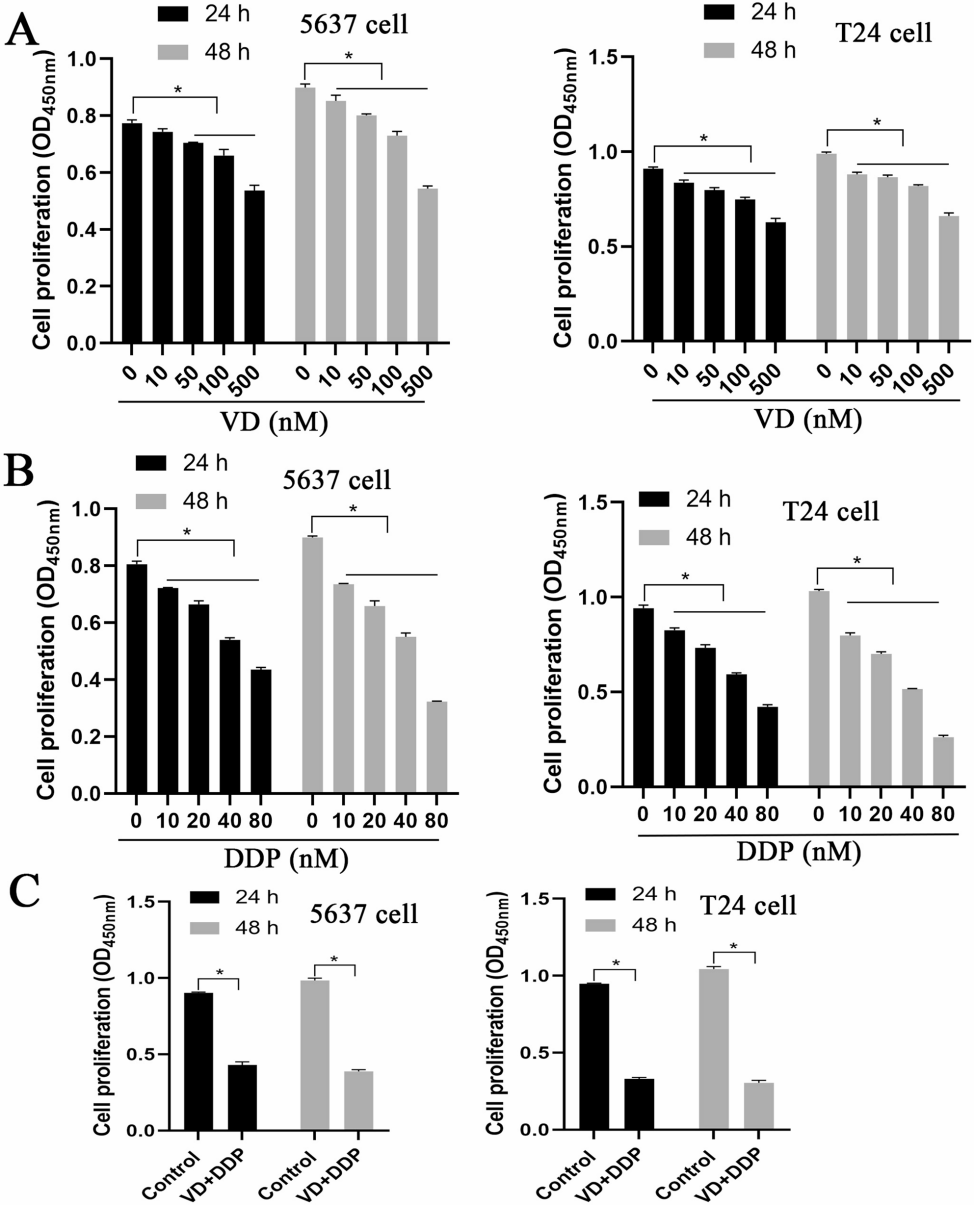
Screening of VD and DDP concentrations. **A**: T24 and 5637 cells were treated with different doses of VD (0, 10, 50, 100, and 500 nM) for 24 and 48 h, and cell proliferation was detected using the CCK-8 assay. **B**: T24 and 5637 cells were treated with different doses of DDP (0, 10, 20, 40, and 80 nM) for 24 and 48 h, and the CCK-8 assay was used to detect cell proliferation. **C**: T24 and 5637 cells were treated with 100 nM VD, 50.901 nM, and 66.968 nM DDP, respectively. After 24 and 48 h of intervention, cell proliferation was detected using the CCK-8 assay

Herein, T24 and 5637 cells were treated with different doses of VD. Compared with VD-untreated cells, treatment with 10, 50, 100, and 500 nM VD significantly reduced the proliferative capacity of T24 and 5637 for 24 and 48 h (Fig. 1A). We selected 100 nM VD for 24 h induction in subsequent experiments. Simultaneously, we treated T24 and 5637 cells with different doses of DDP. Compared with DDP-untreated cells, treatment with 10, 20, 40, and 80 nM DDP significantly reduced the proliferative capacity of T24 and 5637 cells for 24 and 48 h (Fig. 1B). Accordingly, we selected 24-h DDP treatment for subsequent experiments. Based on our calculations, the IC_50_ values for T24 and 5637 cells were 50.901 and 66.968 nM, respectively. Next, T24 and 5637 cells were treated with 100 nM VD and 50.901 and 66.968 nM DDP, respectively. Compared with that of the control group, the proliferative ability of the VD + DDP group was significantly reduced in both T24 and 5637 cells (Fig. 1C). VD-sensitive T24 cells were selected for subsequent experiments.

### Effects of VD combined with DDP on cell proliferation and apoptosis

**Fig. 2:**
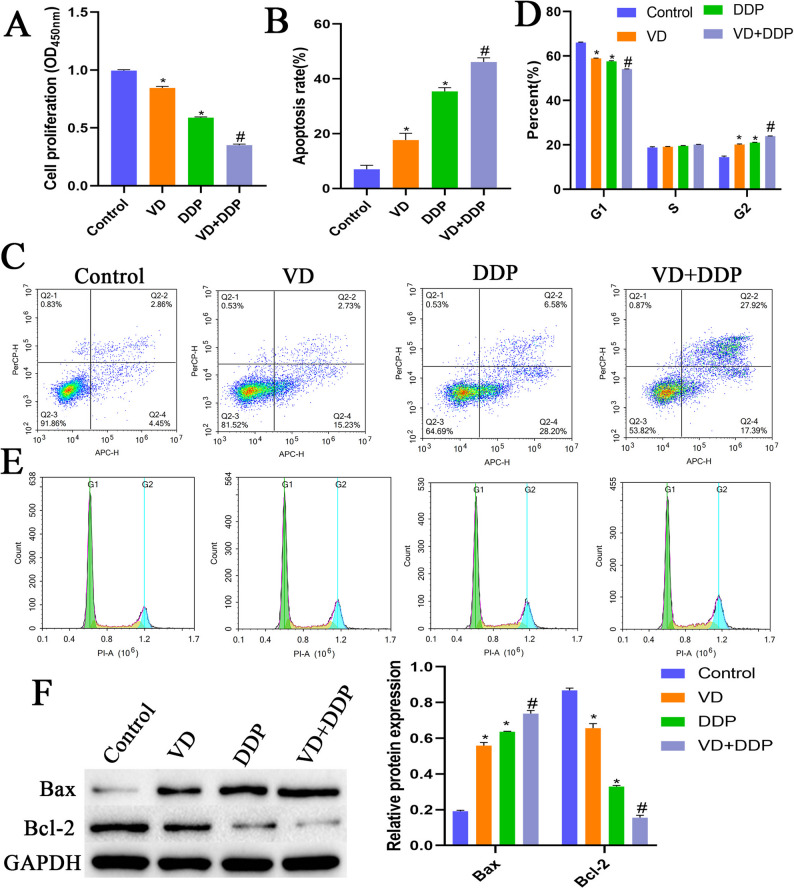
Effects of VD combined with DDP on cell proliferation and apoptosis. **A**: The CCK8 assay was used to assess cell proliferation in each group. **B**-**C**: Flow cytometry was performed to detect the cellular apoptosis rate in each group. **D**-**E**: Flow cytometry was performed to detect the cell cycle status of each group. F: Western blot analysis to detect cellular expression of Bax and Bcl2 proteins in each group. *P < 0.05, compared with the control group; #*P* < 0.05, compared with the VD and DDP groups

Compared with the control group, the VD and DDP groups exhibited significantly reduced proliferation capacities and significantly enhanced apoptotic rates. Compared with the VD and DDP groups, the VD + DDP group demonstrated a significantly reduced proliferation capacity and a significantly elevated apoptotic rate (Fig. 2A-C). Meanwhile, the VD and DDP groups showed an increased proportion of cells in the G2 phase and a decreased proportion of cells in the G1 phase compared with the control group. Compared with the VD and DDP groups, the VD + DDP group exhibited an increased proportion of cells in the G2 phase and a decreased proportion of cells in the G1 phase (Fig. 2D-E). Compared with the control group, the VD and DDP groups exhibited significantly upregulated expression of Bax, while that of Bcl-2 was significantly downregulated. Compared with the VD and DDP groups, the VD + DDP group displayed significantly upregulated Bax expression, along with significantly downregulated Bcl-2 expression (Fig. 2F). These results suggested that VD combined with DDP could suppress cell proliferation and promote apoptosis.

### Effect of VD combined with DDP on the Warburg effect

**Fig. 3:**
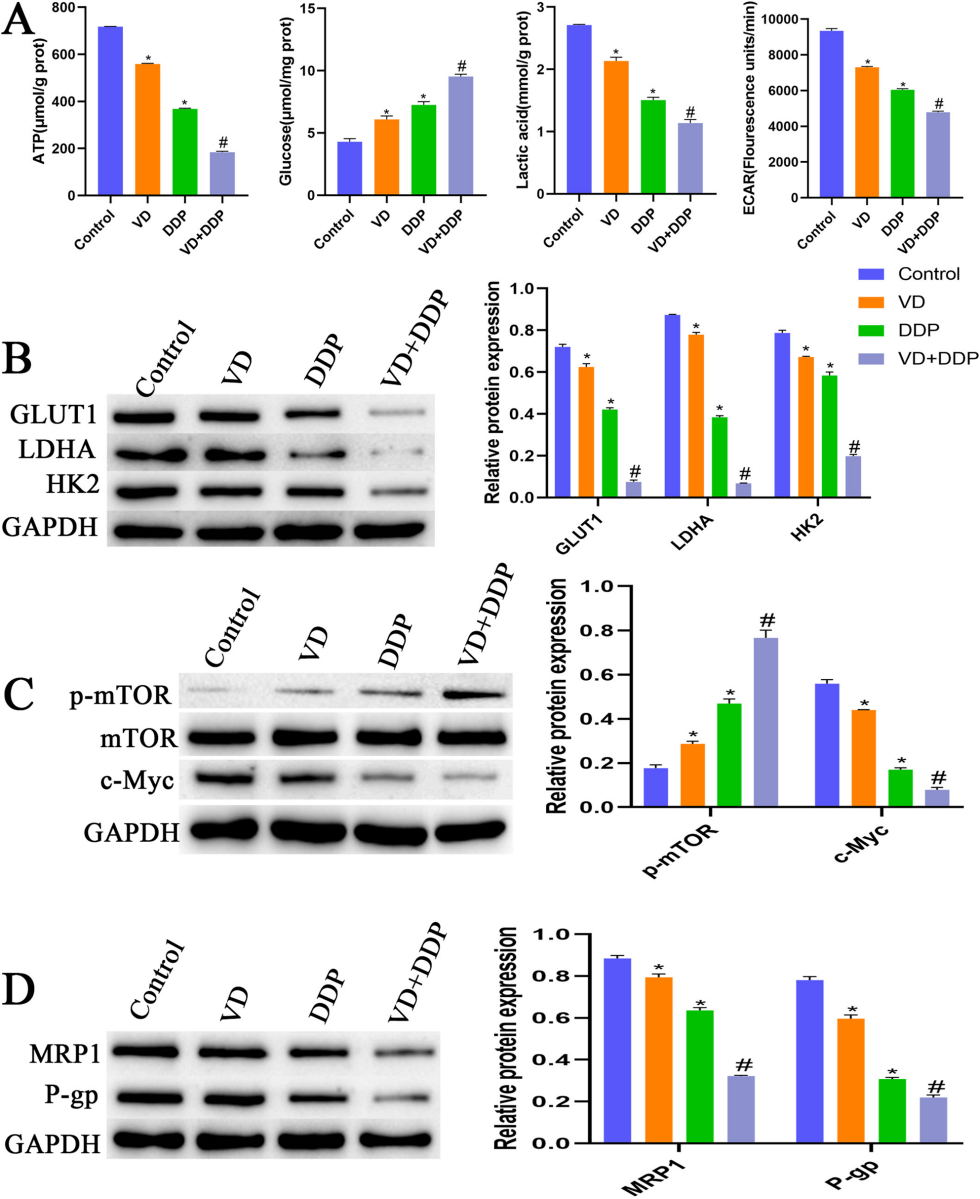
Effect of VD combined with DDP on the Warburg effect in cells. **A**: Biochemical detection of ATP production, glucose uptake, lactate levels and ECAR.in cells in each group. **B**: Western blot analysis of expression of Warburg-related proteins GLUT1, LDHA, and HK2 in cells. **C**: Western blot analysis of protein expression levels of Warburg upstream glycolysis-related proteins p-mTOR, mTOR, and c-Myc in cells. **D**: Western blot analysis of multidrug resistance protein 1 (MRP1) and multidrug resistance gene type 1 p-glycoprotein (P-gp) expression in cells. *P < 0.05, compared with the control group; #*P* < 0.05, compared with the VD and DDP groups

As shown in Fig. 3A, compared with cells in the control group, cells in the VD and DDP groups exhibited significantly reduced ATP, ECAR, and lactic acid contents, while the glucose content was significantly increased. Compared with cells in the VD and DDP groups, cells in the VD + DDP group exhibited significantly reduced ATP, ECAR, and lactic acid contents and a significantly elevated glucose content. Compared with cells in the control group, cells in the VD and DDP groups demonstrated significantly downregulated protein expression of GLUT1, LDHA, HK2, c-Myc, MRP1, and P-gp and significantly upregulated p-mTOR protein expression. Compared with cells in the VD and DDP groups, cells in the VD + DDP group exhibited significantly downregulated protein expression of GLUT1, LDHA, HK2, c-Myc, MRP1, and P-gp, while that of p-mTOR protein was significantly upregulated (Fig. 3B-D). These results indicated that VD combined with DDP could suppress the Warburg effect in cells and reduce drug resistance.

### VD combined with DDP promotes cell apoptosis by inhibiting the Warburg effect

**Fig. 4:**
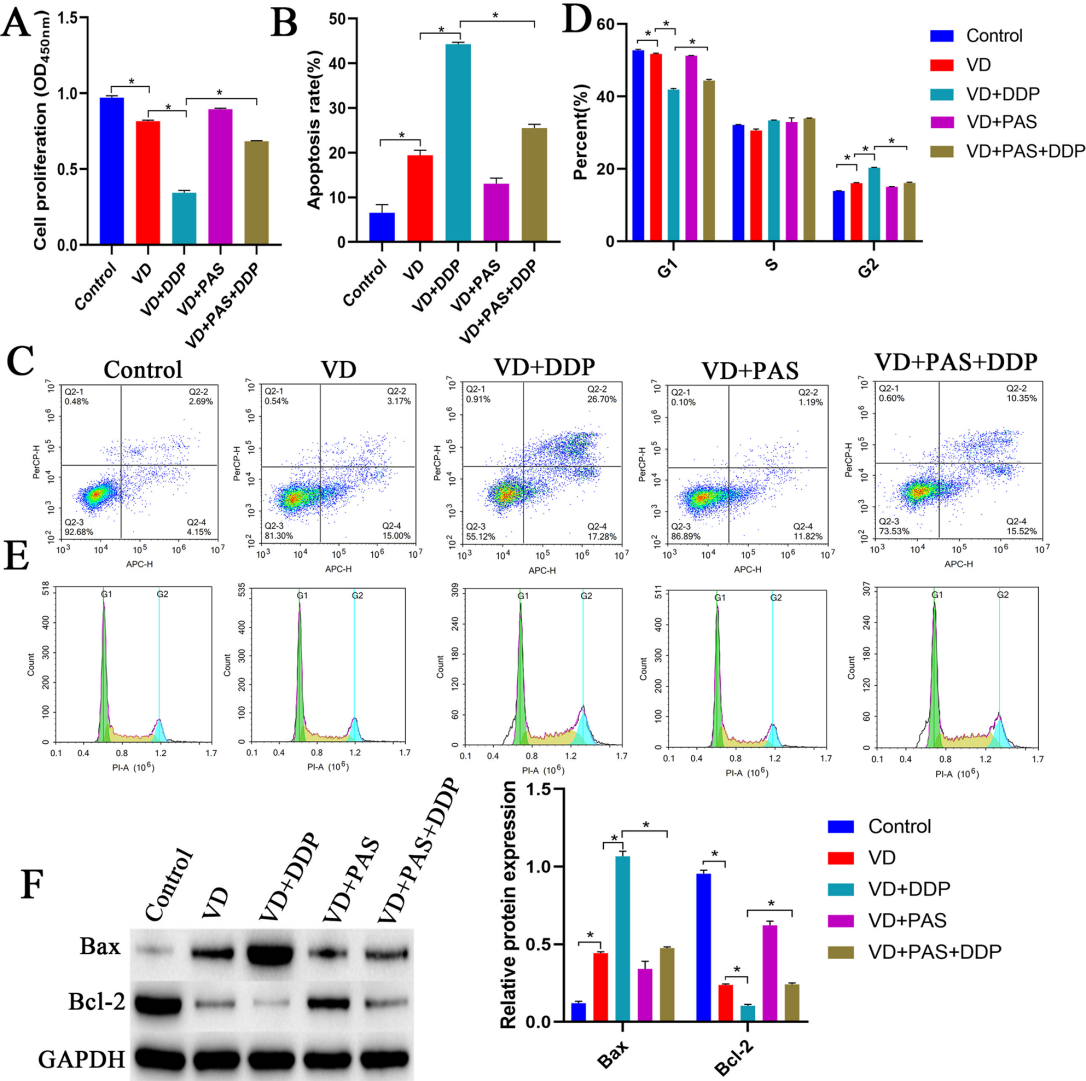
VD combined with DDP promotes cell apoptosis by inhibiting the Warburg effect. **A**: The CCK8 assay was used to detect cell proliferation in each group. **B**-**C**: Flow cytometry was performed to detect the cellular apoptosis rate in each group. **D**-**E**: Flow cytometry was performed to detect changes in the cell cycle in each group. **F**: Western blot analysis to detect Bax and Bcl2 protein expression in cells in each group

To further verify whether VD combined with DDP inhibited cell proliferation and promoted apoptosis by inhibiting the Warburg effect, we treated cells with PAS. Compared with the control group, the VD group had a significantly reduced proliferative capacity and cell proportion in the G1 phase, accompanied by a significantly elevated apoptotic rate and cell proportion in the G2 phase. Compared with the VD group, the VD + DDP group showed a significantly decreased proliferative capacity and cell proportion in the G1 phase, with a significantly increased apoptotic rate and cell proportion in the G2 phase. Compared with the VD + PAS + DDP group, the VD + DDP group exhibited significantly reduced proliferative capacity and a lower cell proportion in the G1 phase, along with a significantly elevated apoptotic rate and a higher cell proportion in the G2 phase (Fig. 4A-E). Compared with the control group, the VD group exhibited significantly elevated Bax expression and significantly reduced Bcl-2 expression. Compared with the VD group, the VD + DDP group displayed significantly increased Bax expression and significantly decreased Bcl-2 expression. Compared with the VD + PAS + DDP group, the VD + DDP group showed significant upregulation of Bax expression and significant downregulation of Bcl-2 expression (Fig. 4F). These results suggested that VD combined with DDP could inhibit cell proliferation and promote apoptosis by impacting the Warburg effect.

### VD combined with DDP inhibits the Warburg effect

**Fig. 5:**
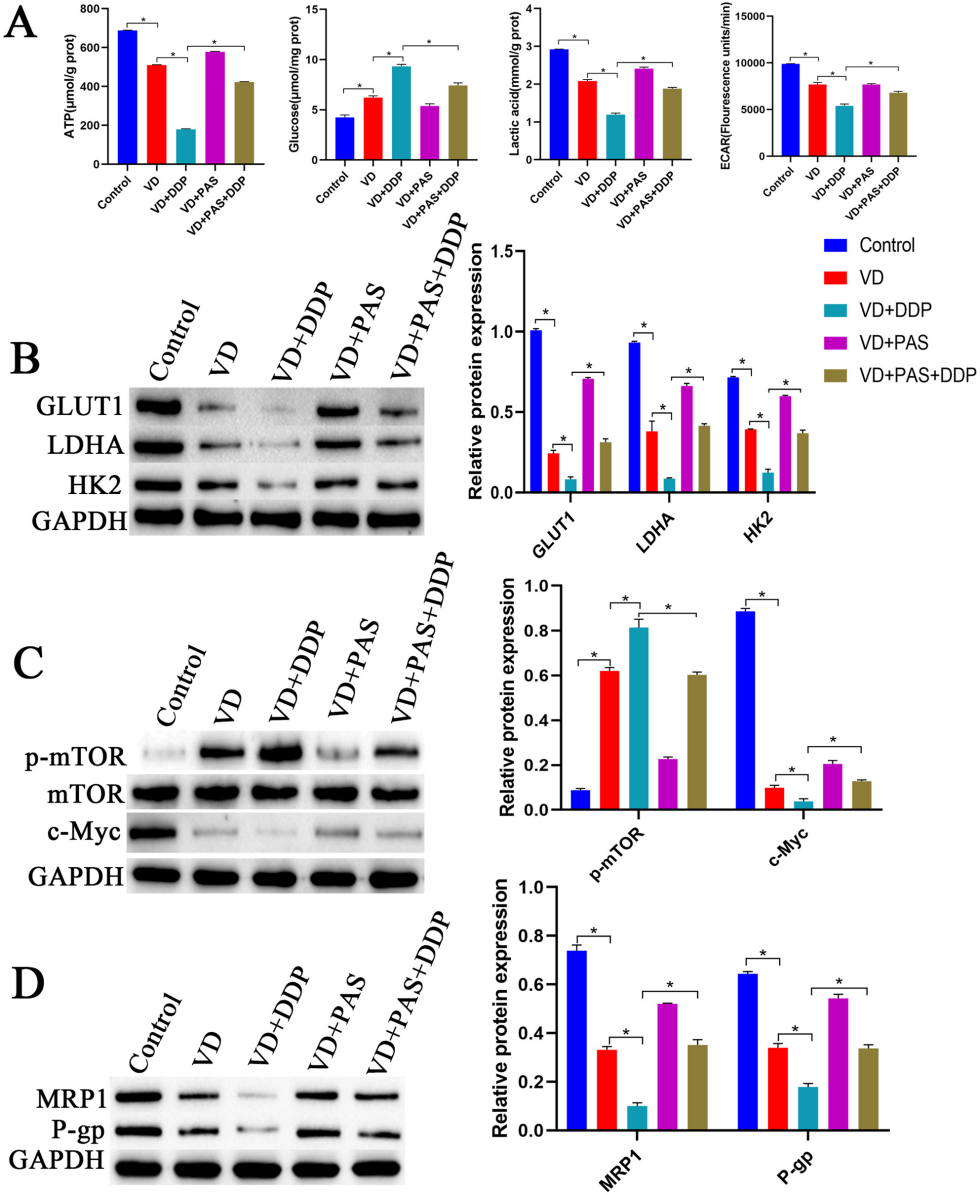
VD combined with DDP can inhibit the Warburg effect. **A**: Biochemical detection of cellular ATP production, glucose uptake, lactate levels and ECAR. **B**: Western blot analysis to detect protein expression levels of Warburg-related proteins GLUT1, LDHA, and HK2 in cells. **C**: Western blot analysis to detect protein expression levels of Warburg upstream glycolysis-related proteins p-mTOR, mTOR, and c-Myc in cells. **D**: Western blot analysis to detect the expression of multidrug resistance protein 1 (MRP1) and multidrug resistance gene type 1 p-glycoprotein (P-gp) in cells

As shown in Fig. 5A, ATP and lactate levels in the VD group were significantly reduced, while the glucose level was significantly elevated. Compared with the VD group, the VD + DDP group exhibited significantly reduced cellular ATP, ECAR, and lactic acid contents and a significantly elevated glucose level. Compared with the VD + PAS + DDP group, the VD + DDP group showed a significant reduction in ATP, ECAR, and lactic acid contents, with a significantly elevated glucose content. Compared with the control group, the VD group displayed significantly downregulated protein expression of GLUT1, LDHA, HK2, c-Myc, MRP1, and P-gp and significantly upregulated p-mTOR protein expression. Compared with the VD group, the VD + DDP group showed significantly downregulated protein expression of GLUT1, LDHA, HK2, c-Myc, MRP1, and P-gp and significantly upregulated p-mTOR protein expression. Compared with the VD + PAS + DDP group, the VD + DDP group displayed significantly downregulated protein expression of GLUT1, LDHA, HK2, c-Myc, MRP1, and P-gp and significantly upregulated p-mTOR protein expression (Fig. 5B-D). These results indicated that VD combined with DDP could suppress the Warburg effect and reduce cellular drug resistance.

### VD combined with DDP inhibits the Warburg effect via the AKT/mTOR pathway

**Fig. 6:**
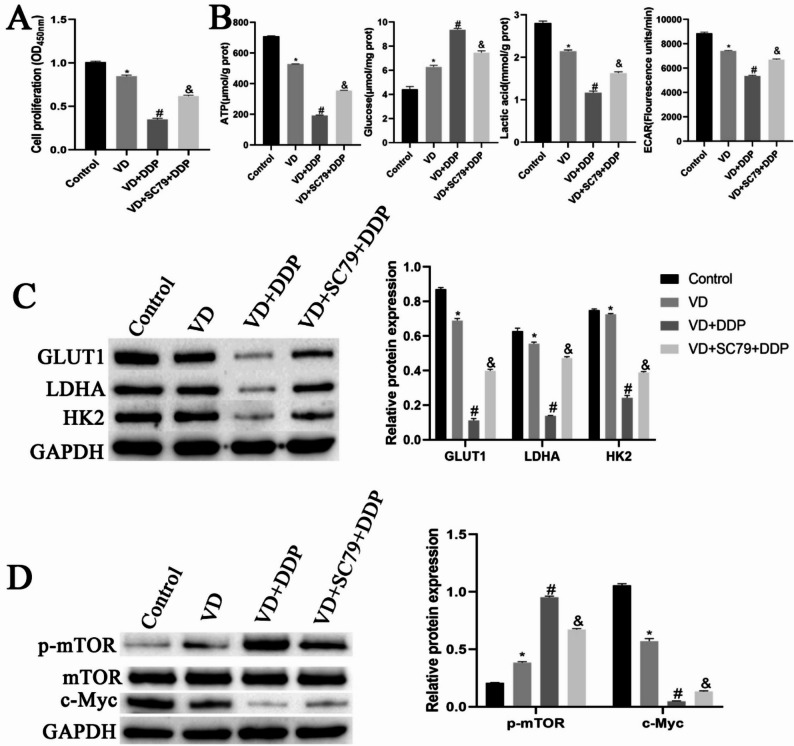
VD combined with DDP inhibits the Warburg effect through the AKT/mTOR pathway. **A**: The CCK8 assay was used to detect cell proliferation in each group. **B**: Biochemical detection of cellular ATP production, glucose uptake, lactate levels and ECAR. **C**: Western blot analysis was performed to detect protein editing levels of Warburg-related proteins GLUT1, LDHA, and HK2 in cells. **D**: Western blot analysis to detect protein expression levels of Warburg upstream glycolysis-related proteins p-mTOR, mTOR, and c-Myc in cells. *P < 0.05, compared with the control group; #*P* < 0.05, compared with the VD group; &*P* < 0.05, compared with the VD + DDP group

Compared with the control group, the VD group exhibited a significantly reduced cellular proliferative capacity. The cellular proliferative ability of the VD + DDP group was significantly reduced compared with that of the VD group. Conversely, the proliferative ability of cells in the VD + SC79 + DDP group was significantly increased compared with that of the VD + DDP group (Fig. 6A). As shown in Fig. 6B, the cellular ATP, ECAR, and lactic acid content were significantly reduced in the VD group compared with those in the control group, whereas the glucose content was significantly elevated. Compared with the VD group, the VD + DDP group exhibited significantly reduced cellular ATP, ECAR, and lactic acid contents and a significantly elevated glucose content. Compared with the VD + DDP group, the VD + SC79 + DDP group exhibited significantly elevated ATP and lactic acid content, as well as a significantly reduced glucose content. Compared with the control group, the VD group displayed significantly downregulated protein expression of GLUT1, LDHA, HK2, and c-Myc and significantly upregulated p-mTOR protein expression. Compared with the VD group, the VD + DDP group exhibited significantly downregulated protein expression of GLUT1, LDHA, HK2, and c-Myc and significantly upregulated p-mTOR protein expression. Compared with the VD + DDP group, the VD + SC79 + DDP group displayed significantly upregulated protein expression of GLUT1, LDHA, HK2, and c-Myc and significantly downregulated p-mTOR protein expression (Fig. 6C-D). These results indicated that VD combined with DDP could inhibit the Warburg effect via the AKT/mTOR pathway.

## Discussion

Patients with advanced BCa exhibit a limited response to DDP treatment, with approximately 30‒50% of patients failing to receive beneficial treatment [22, 23]. Approximately 50% of the patients who undergo cystectomy develop metastatic disease within 2 years. Therefore, there is an urgent need for new methods to improve the efficacy of DDP and patient prognosis. In this study, we demonstrated that VD and DDP could suppress BCa cell proliferation and the Warburg effect, promote apoptosis, and activate the AKT/mTOR pathway. Accordingly, VD may promote the apoptosis of BCa cells and enhance DDP sensitivity by inhibiting the Warburg effect through the AKT/mTOR pathway.

Reportedly, VD levels were lower in patients with DDP-resistant BCa than in those with DDP-sensitive BCa [24]. VD has a broad spectrum of antitumor effects and can additively or synergistically enhance the antitumor activity of DDP [25]. Hsu et al. [26] reported that VD could enhance the BCG-mediated anti-BCa immune response, thereby prolonging the survival of BCa mice. However, reports on the combined therapeutic effects of VD and DDP and the potential mechanism of action are lacking. Herein, we found that VD suppressed BCa cell proliferation, promoted apoptosis, and inhibited the expression of drug-resistant genes, with the combination of VD and DDP exhibiting the greatest efficacy. Low levels of vitamin D were found to be associated with an elevated risk of various cancers, including colon, breast, prostate, and blood cell cancer [27]. Liu et al. [28] found that vitamin D could suppress colorectal cancer cell proliferation, migration, and invasion by downregulating the Notch1 pathway. Huang et al. [29] found that vitamin D promoted sensitivity to DDP in oral squamous cell carcinoma by inhibiting NF-κB pathway activation. Our study demonstrated that VD could improve the effectiveness of DDP treatment, providing a theoretical basis for improving the overall survival of patients with BCa. Furthermore, combination therapies (multiple drugs targeting the same cellular pathway to produce synergistic effects and achieve greater therapeutic effects) have been used clinically. In this study, we demonstrated that combining VD and DDP could exert a better therapeutic effect than single therapy, which has considerable clinical relevance.

The Warburg effect is a characteristic metabolic phenotype of tumor cells that sustains their growth and survival [30]. SMAR1 has been shown to inhibit BCa progression by inhibiting glucose consumption, lactate, ATP production, and glycolytic fluxes and suppressing GLUT1 expression, thereby attenuating the Warburg effect in BCa cells [31]. GLUT1, a member of the cell surface glucose transporter family, plays a role in transporting glucose across cell membranes, thereby promoting tumor cell proliferation and metastasis [32]. c-Myc functions as a key regulator of the Warburg effect by directly activating several glycolytic genes, including LDHA and PKM2 [33, 34]. However, the role of VD in mediating the Warburg effect in BCa cells remains unclear. In this study, VD downregulated the cellular expression of GLUT1, LDHA, HK2, and c-Myc, reduced glucose consumption, and decreased lactate and ATP production. These data suggest that VD promoted the apoptosis of BCa cells by inhibiting the Warburg effect. Accordingly, cells were treated with PAS to further validate the role of VD on the Warburg effect in BCa cells. VD reversed the action of PAS on bladder cancer cells by suppressing the Warburg effect. This is similar to previous findings showing that vitamin D can suppress aerobic glycolysis in colorectal cancer cells by degrading c-Myc [35]. Xu et al. [36] reported that vitamin D regulated primitive cell metabolism in acute myeloid leukemia by blocking the Warburg effect. Yiyan et al. [37] employed in vivo and in vitro experiments to demonstrate that VD impacted the Warburg effect in non-small cell lung cancer cells by regulating the PI3K/AKT/mTOR signaling pathway, thereby inhibiting the development of cancer.

PI3K and Akt, upstream factors of mTOR, are suitable therapeutic targets for various cancers [38, 39]. The PI3K/Akt signaling pathway plays a crucial role in regulating several key cellular processes, including cell proliferation, energy homeostasis, and survival. mTOR, a downstream effector molecule of the PI3K/Akt pathway, is a therapeutic target for various cancers. VD reportedly affects the Warburg effect and stemness maintenance in non-small cell lung cancer cells by regulating the PI3K/AKT/mTOR signaling pathway [37]. In addition, in vivo experiments revealed that VD ameliorated hepatic lipid disorders in type 2 diabetic mice by activating autophagy and regulating AMPK/Akt-mTOR signaling [40]. Almaiman et al. [41] found that the combined treatment with VD, 5-fluorouracil, and metformin inhibited tumor progression in colorectal cancer mice by regulating the Akt/mTOR pathway. The AKT/mTOR signaling pathway acts as an intermediate junction in tumor cells and can be regulated by the proto-oncogenes Ras or Src, as well as by Myc activity [42]. However, whether the roles of VD and DDP in BCa are related to the AKT/mTOR pathway is yet to be established. In the present study, VD and DDP upregulated p-mTOR expression and reversed the effects of SC79 on BCa cells. Although our results have not been verified in vivo, they suggest that VD and DDP may inhibit the Warburg effect via the AKT/mTOR pathway. In addition, this study still has certain limitations and has not been validated in normal cells. However, previous studies have reported that VD has a relatively small impact on healthy HUVEC cell lines, manifested as no effect on cell proliferation [43].Therefore, in future research, we will design experiments to further validate this part.

## Conclusion

In conclusion, using in vitro experiments, this study verified that VD can inhibit BCa cell proliferation, promote apoptosis, suppress the Warburg effect, and reduce drug resistance. Further investigation of the underlying mechanisms suggested that the observed effects may be related to activation of the AKT/mTOR pathway. Therefore, VD levels could serve as biomarkers reflecting the degree of DDP resistance in patients with BCa during clinical treatment. Nevertheless, this study has certain limitations. In future research, we will conduct experiments in normal bladder epithelial cell lines to evaluate the effects of high-concentration VitD treatment similar to that used in this study on cells, and directly compare the results with cancer cell data. In vivo experiments will also be involved for verification. However, our study provided experimental evidence and a mechanistic basis for VD in enhancing BCa chemotherapy sensitivity and promoting combination therapy with DDP, thereby offering a new theoretical foundation for the clinical treatment of BCa.

## Supplementary Information

Supplementary Material 1.

Supplementary Material 2.

## Data Availability

The data used to support the findings of this study have been included in this article.

## Abbreviations

BCa: Bladder cancer
DDP: Cisplatin
VD: 1,25-Dihydroxyvitamin D3
GLUT1: Glucose transporter protein 1
HK2: Hexokinase 2
LDHA: Lactate dehydrogenase A
MRP1: Multidrug resistance protein 1
P-gp: P-glycoprotein
PAS: Pyruvic acid sodium
