# Supplementary figures and images for: Vitamin D promotes apoptosis and enhances cisplatin sensitivity in bladder cancer cells by inhibiting the Warburg effect through the AKT/mTOR pathway
Source: BMC Urol. 2025 Dec 15;26:11. doi: 10.1186/s12894-025-01994-2 (PMC12822197; doi:10.1186/s12894-025-01994-2)

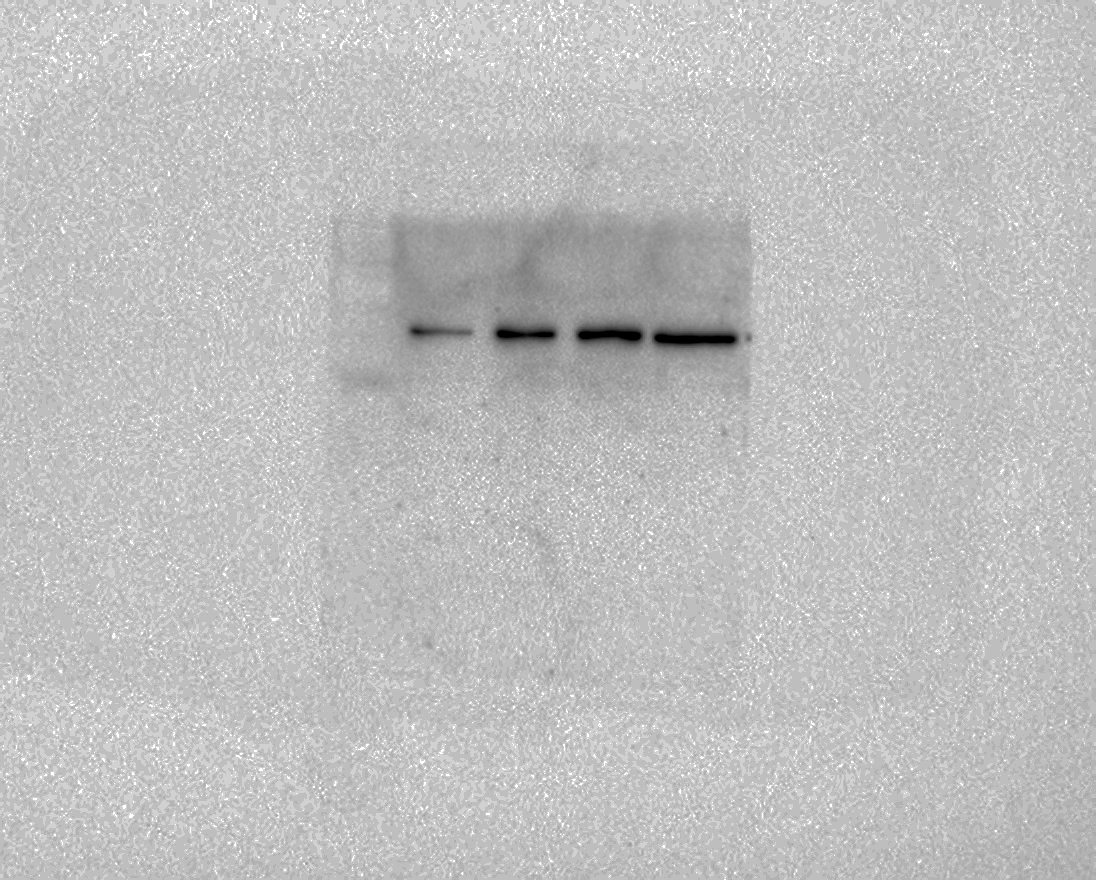

Supplement: Supplementary file 1 — Supplementary Material 1. [file 12894_2025_1994_MOESM1_ESM.zip › Figure 2F/BAX.jpg]

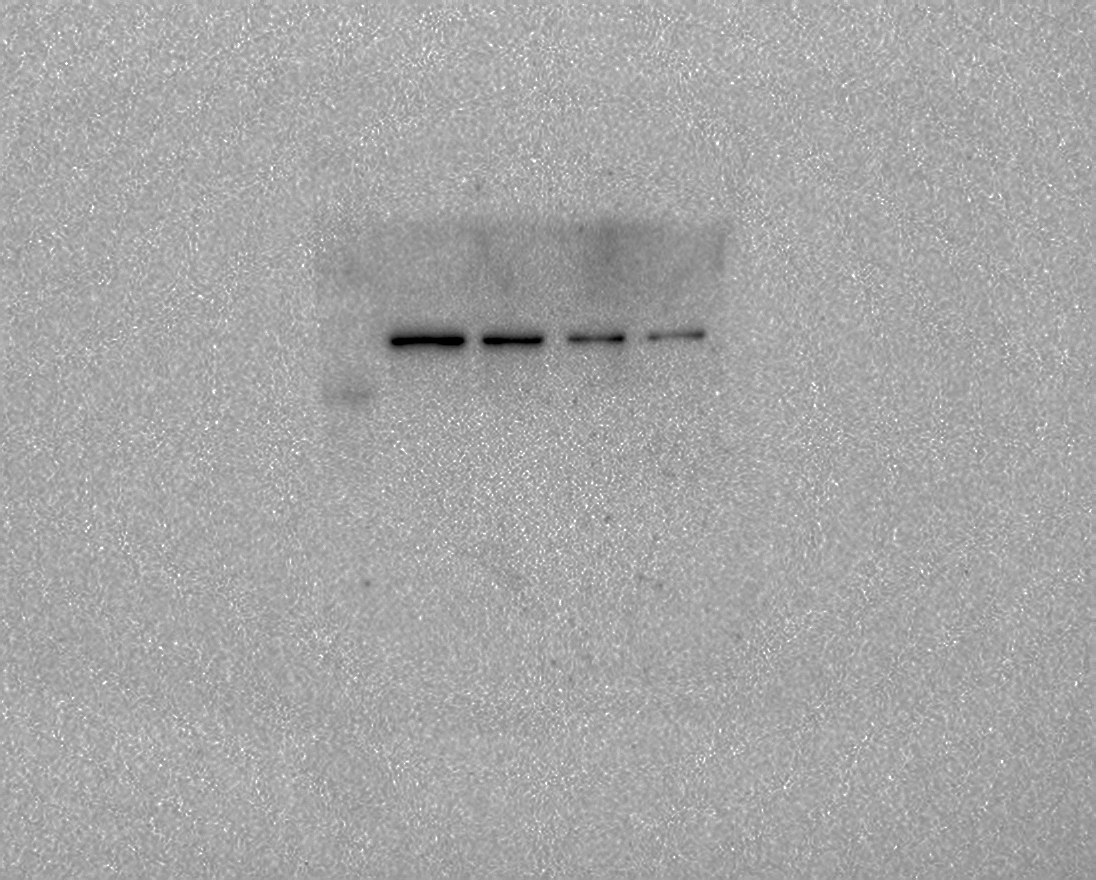

Supplement: Supplementary file 1 — Supplementary Material 1. [file 12894_2025_1994_MOESM1_ESM.zip › Figure 2F/BCL-2.jpg]

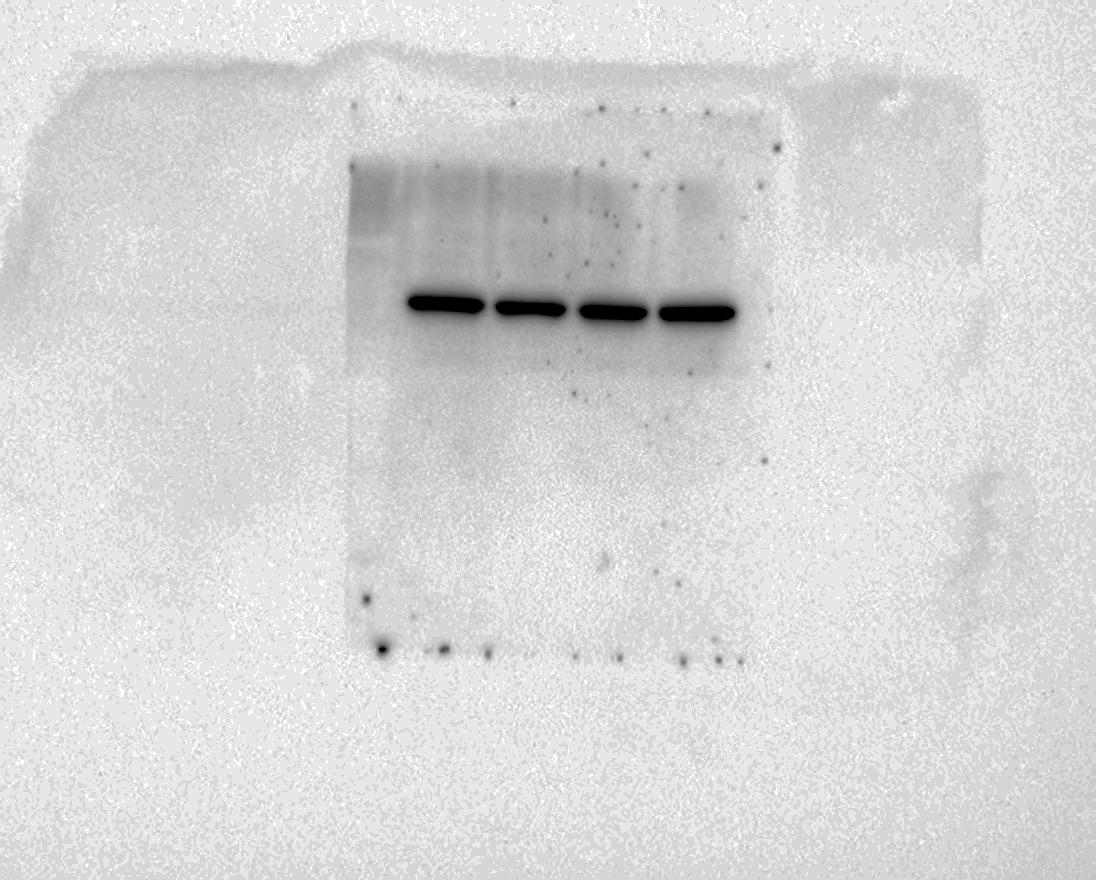

Supplement: Supplementary file 1 — Supplementary Material 1. [file 12894_2025_1994_MOESM1_ESM.zip › Figure 2F/GAPDH.jpg]

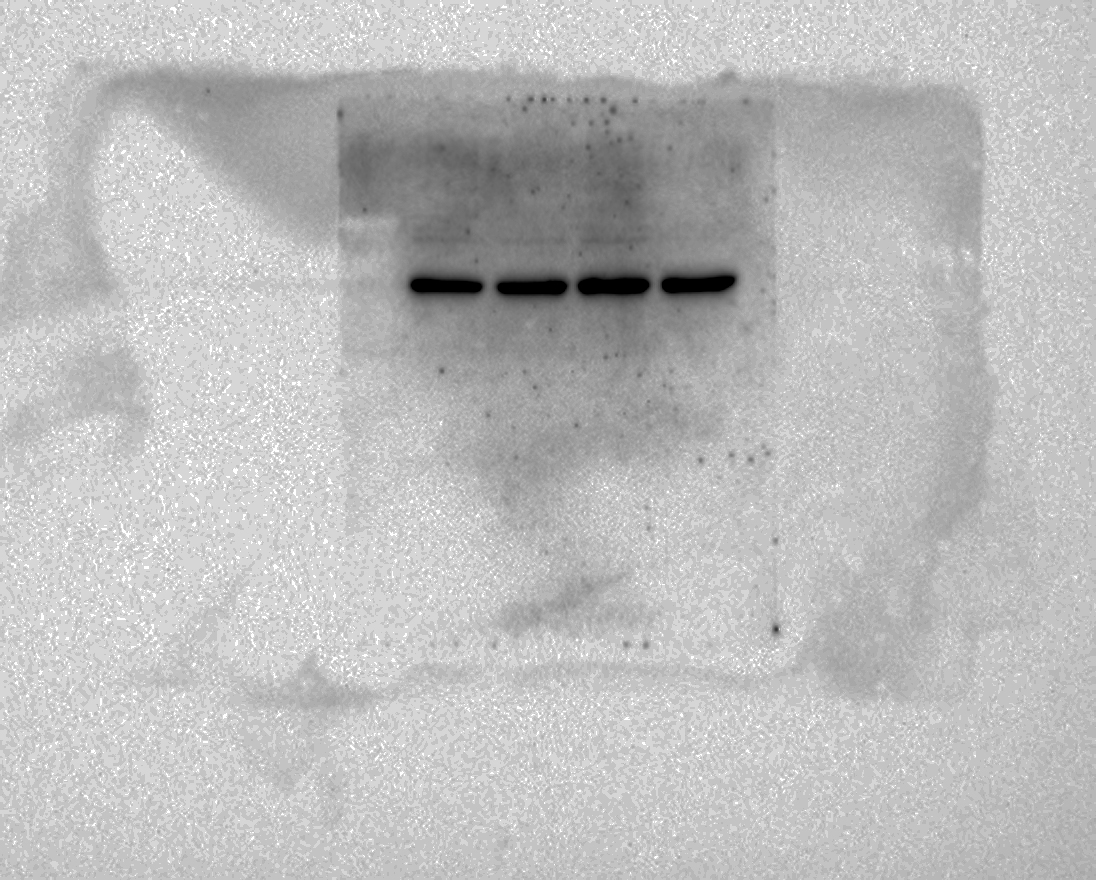

Supplement: Supplementary file 1 — Supplementary Material 1. [file 12894_2025_1994_MOESM1_ESM.zip › Figure 3B/GAPDH.jpg]

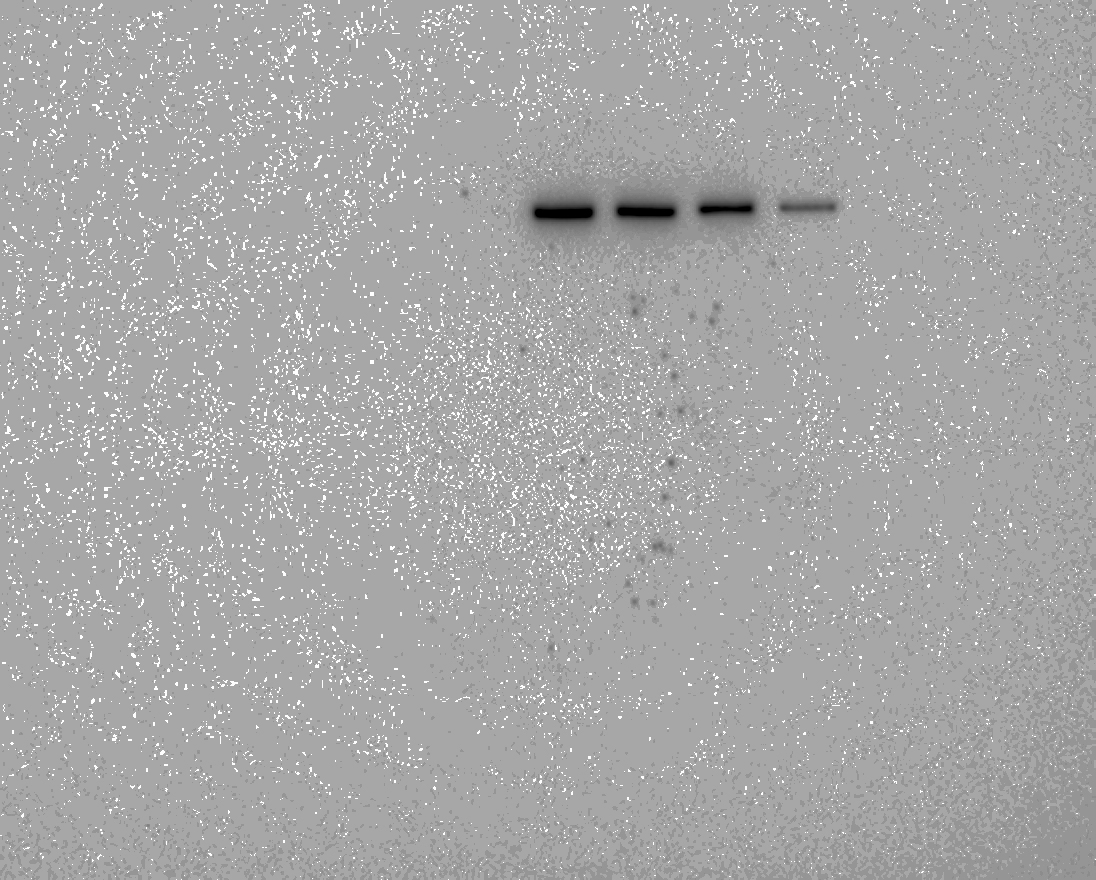

Supplement: Supplementary file 1 — Supplementary Material 1. [file 12894_2025_1994_MOESM1_ESM.zip › Figure 3B/GLUT1.jpg]

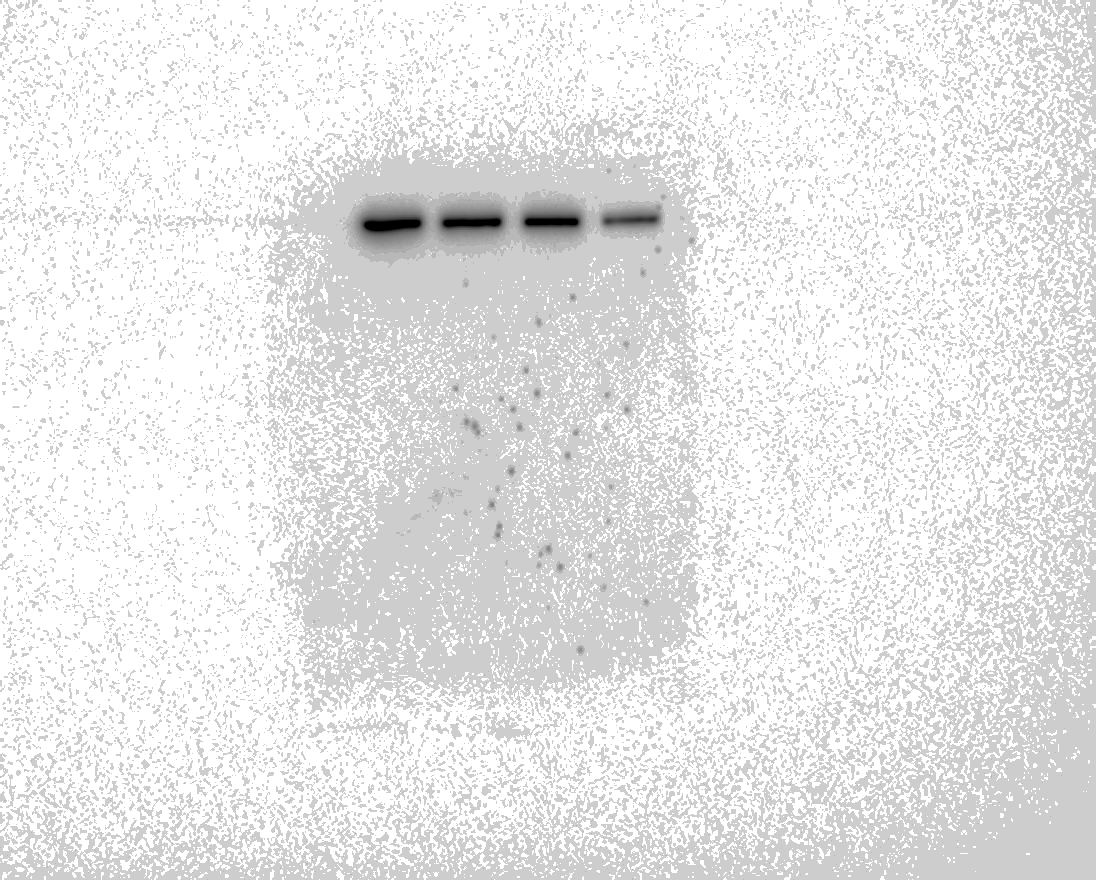

Supplement: Supplementary file 1 — Supplementary Material 1. [file 12894_2025_1994_MOESM1_ESM.zip › Figure 3B/HK2.jpg]

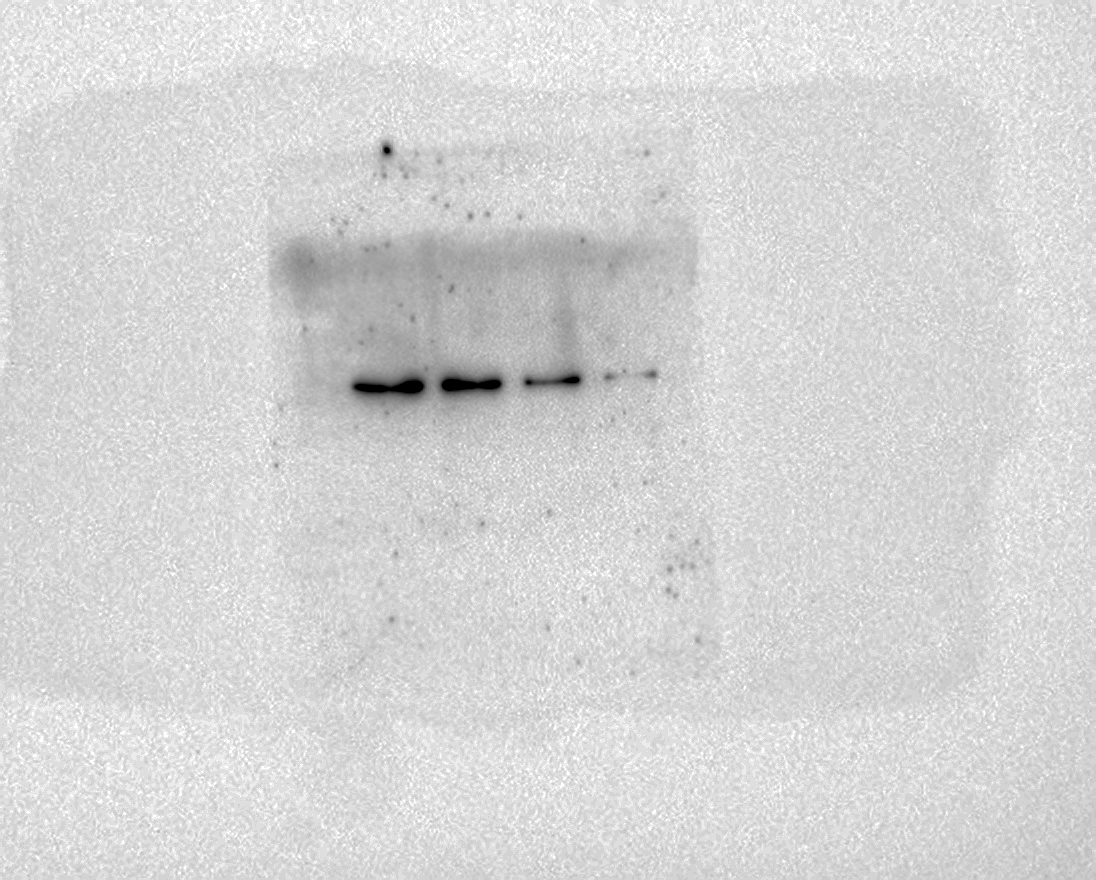

Supplement: Supplementary file 1 — Supplementary Material 1. [file 12894_2025_1994_MOESM1_ESM.zip › Figure 3B/LDHA.jpg]

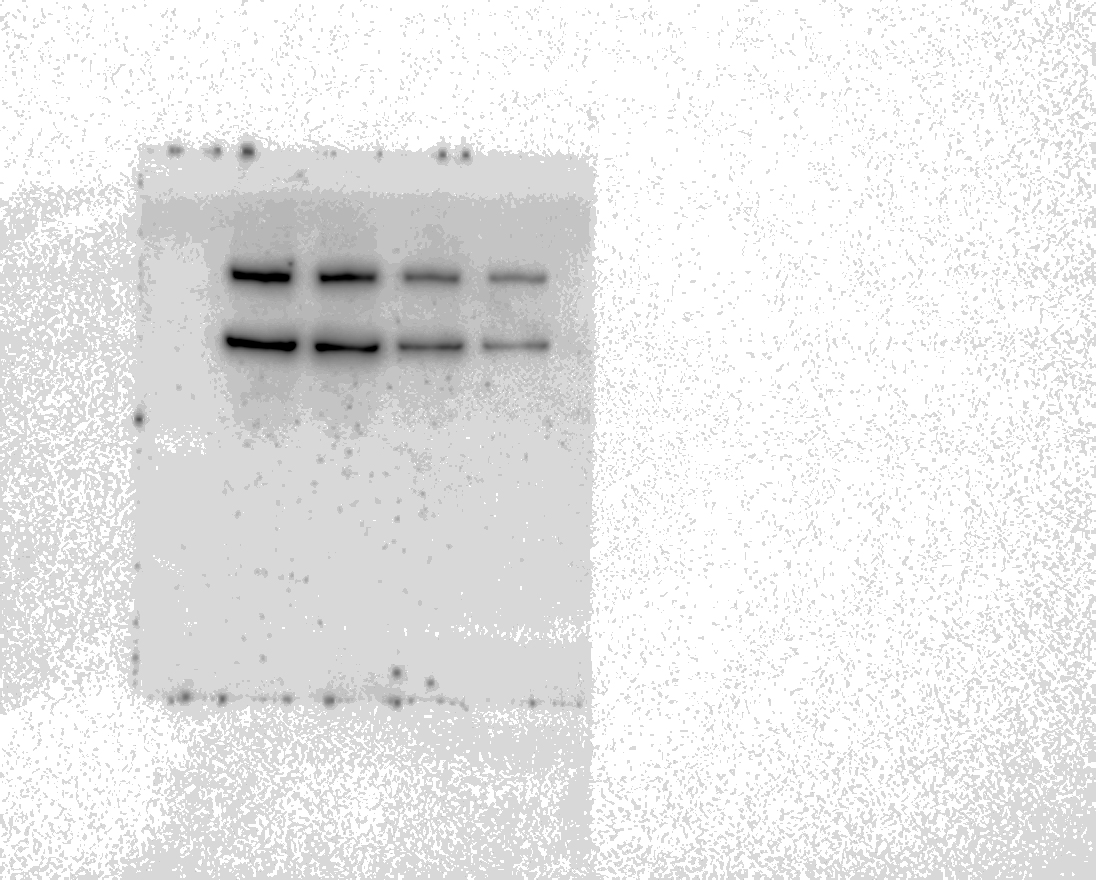

Supplement: Supplementary file 1 — Supplementary Material 1. [file 12894_2025_1994_MOESM1_ESM.zip › Figure 3C/C-MYC.jpg]

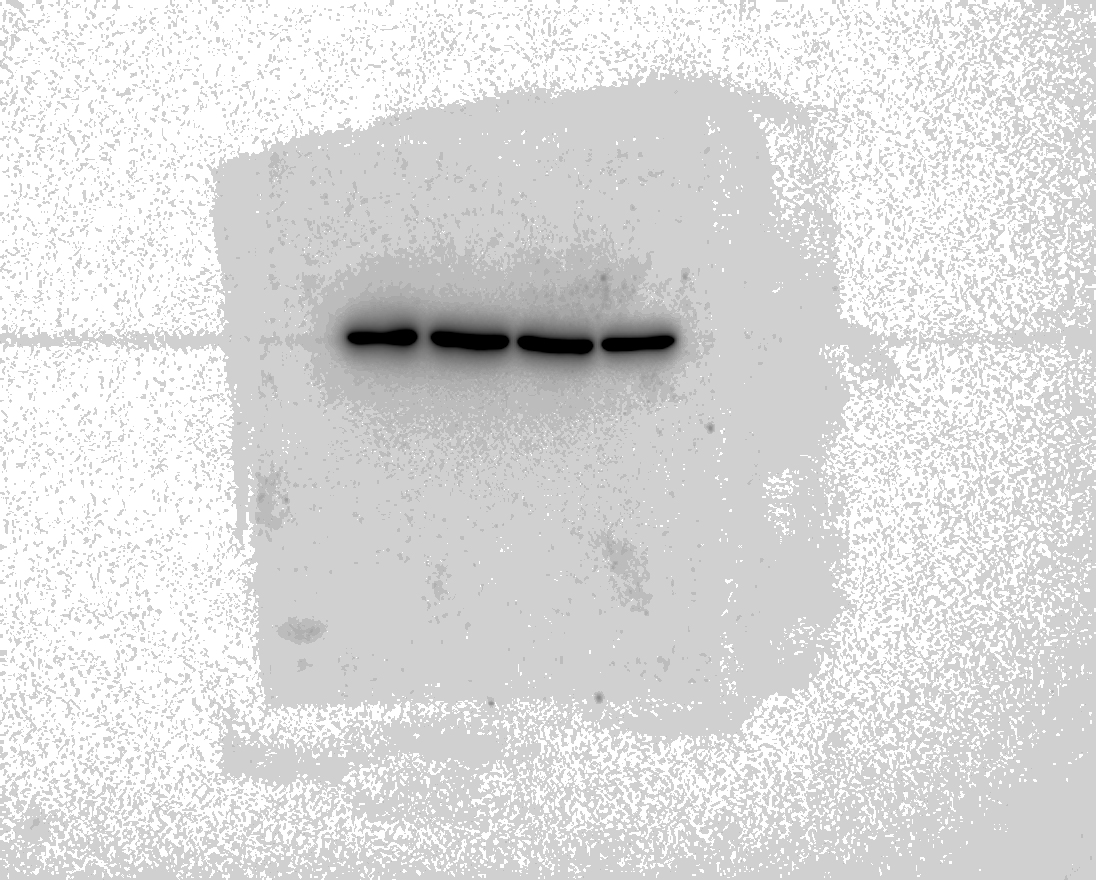

Supplement: Supplementary file 1 — Supplementary Material 1. [file 12894_2025_1994_MOESM1_ESM.zip › Figure 3C/GAPDH.jpg]

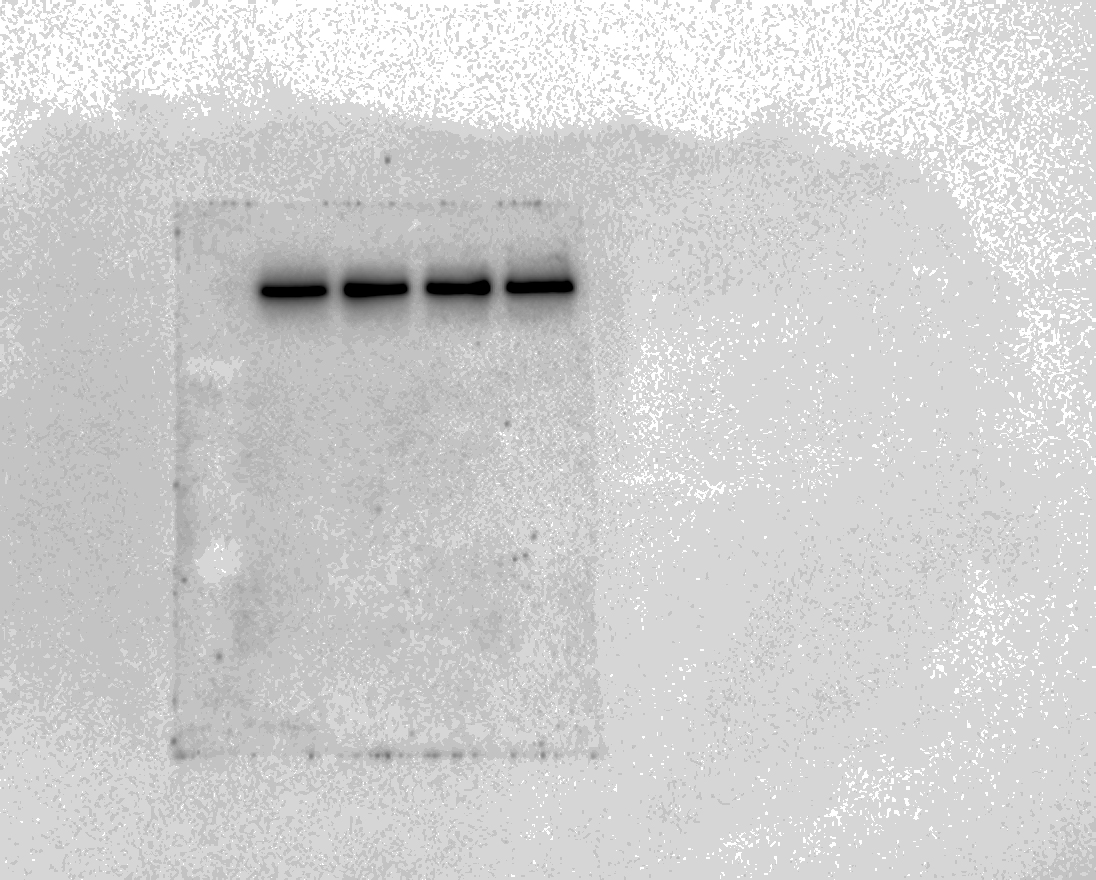

Supplement: Supplementary file 1 — Supplementary Material 1. [file 12894_2025_1994_MOESM1_ESM.zip › Figure 3C/MTOR.jpg]

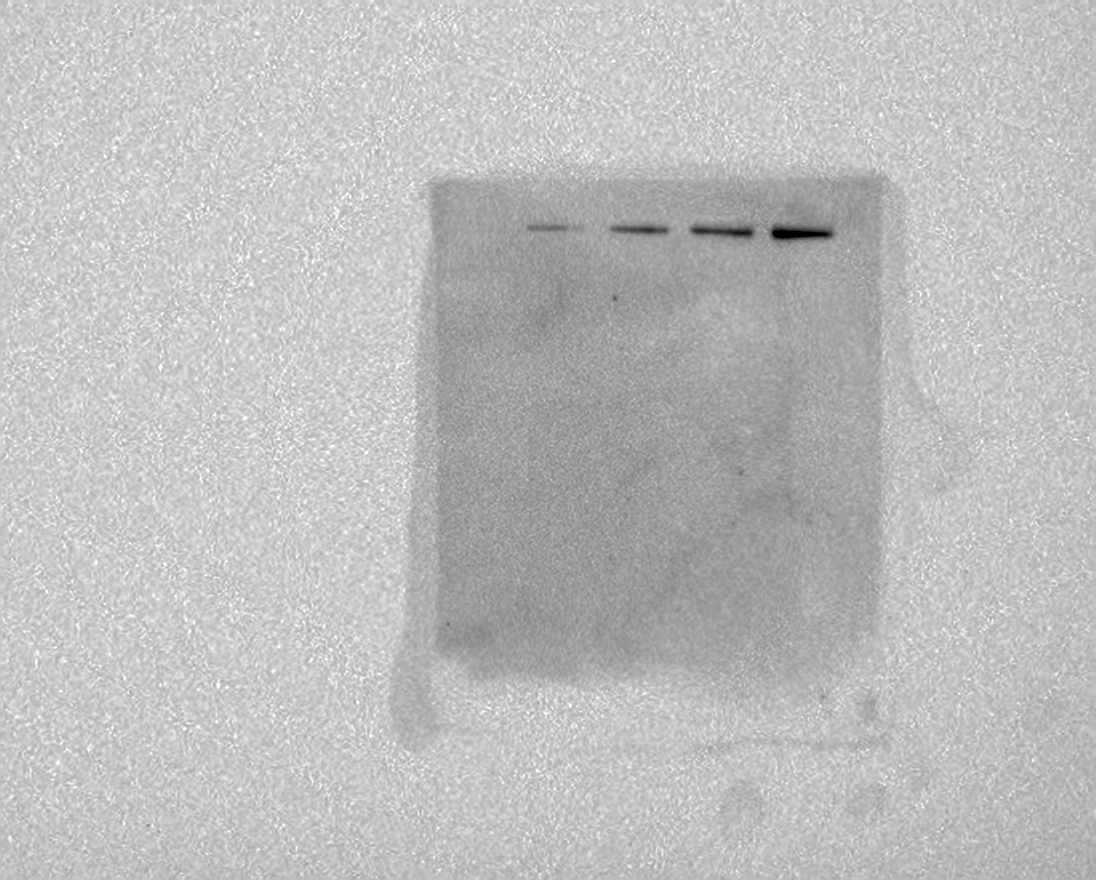

Supplement: Supplementary file 1 — Supplementary Material 1. [file 12894_2025_1994_MOESM1_ESM.zip › Figure 3C/P-mTOR.jpg]

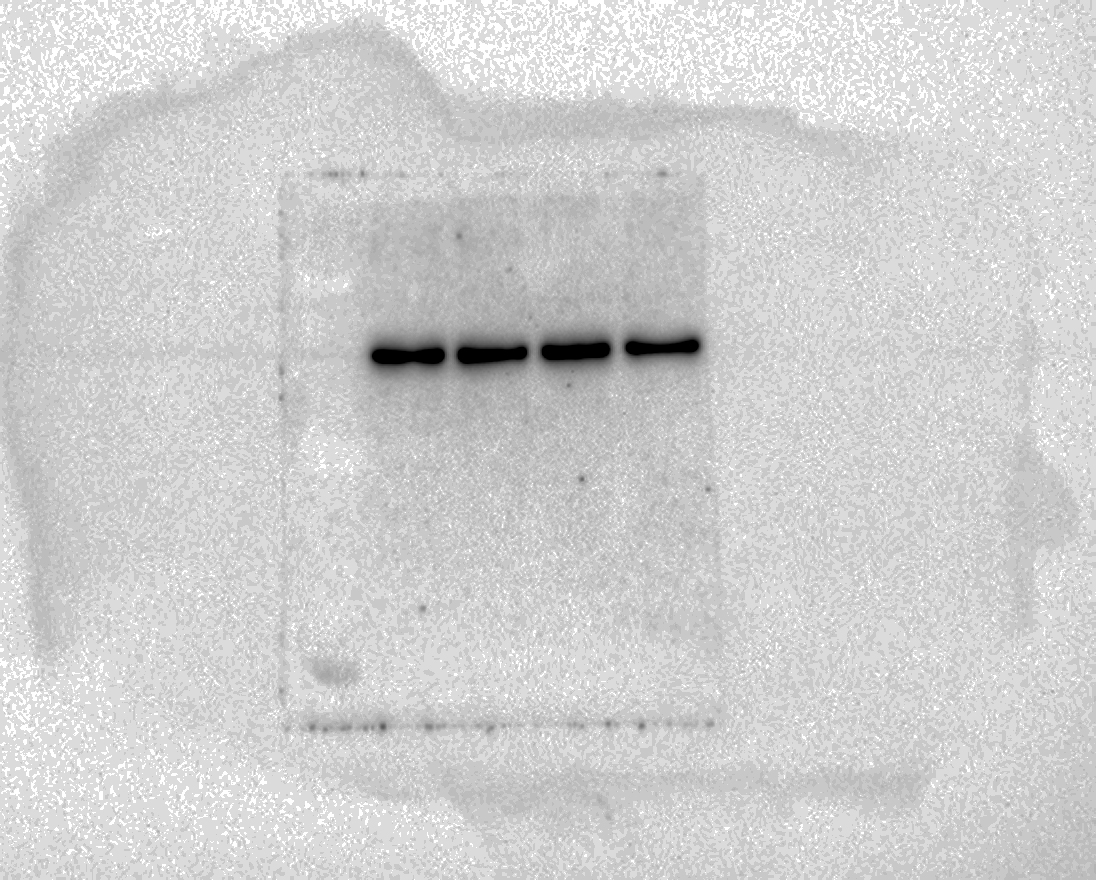

Supplement: Supplementary file 1 — Supplementary Material 1. [file 12894_2025_1994_MOESM1_ESM.zip › Figure 3D/GAPDH.jpg]

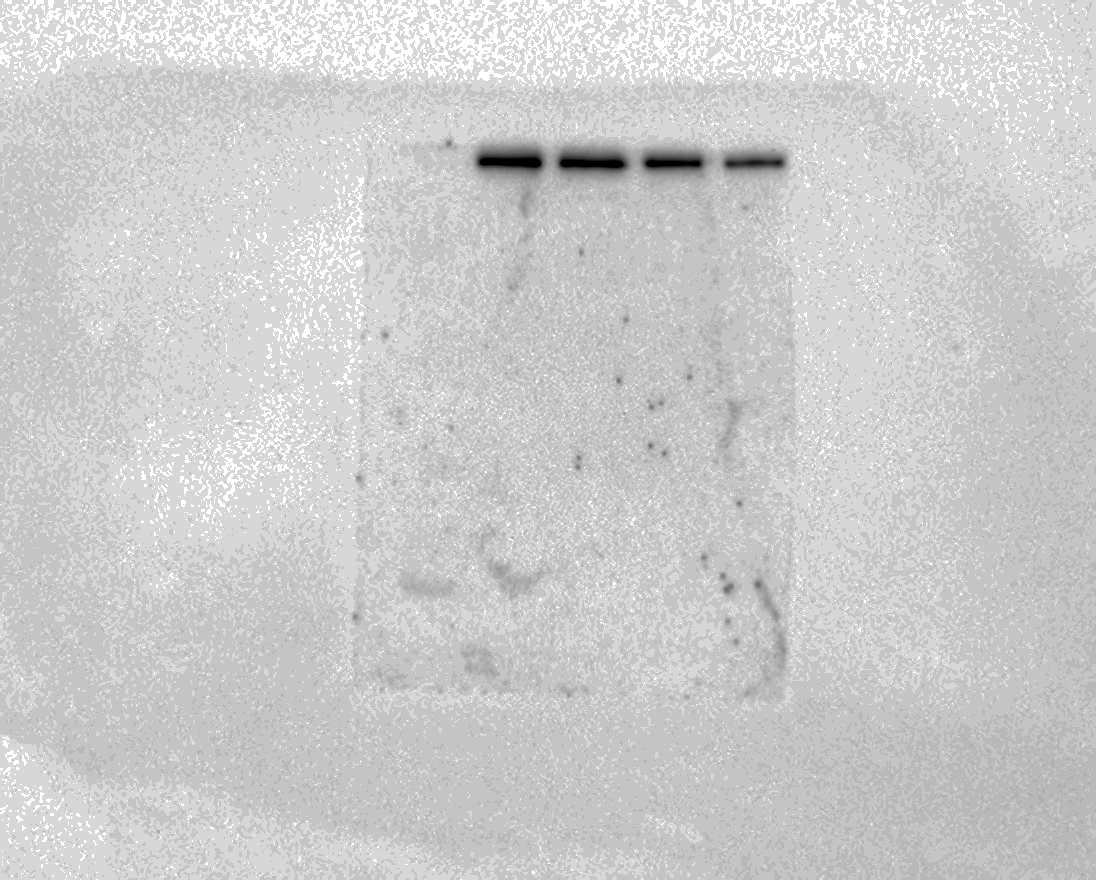

Supplement: Supplementary file 1 — Supplementary Material 1. [file 12894_2025_1994_MOESM1_ESM.zip › Figure 3D/MRP1.jpg]

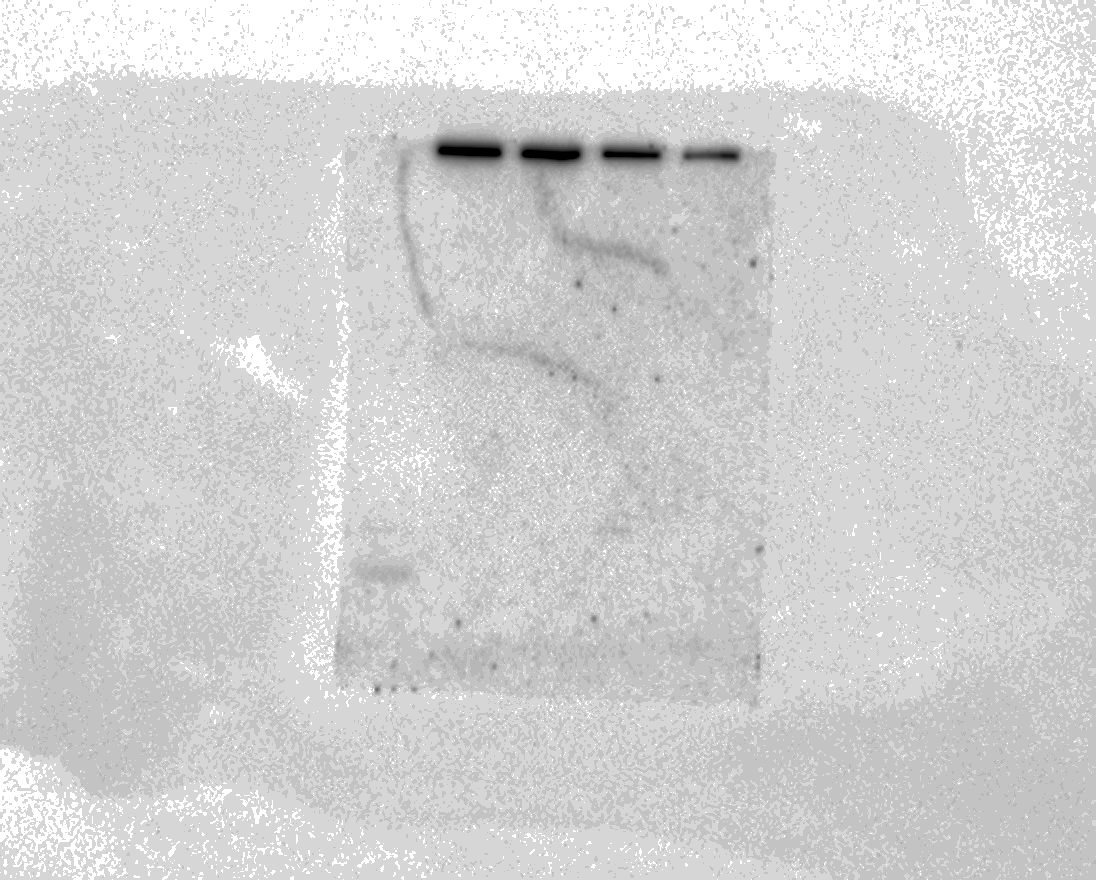

Supplement: Supplementary file 1 — Supplementary Material 1. [file 12894_2025_1994_MOESM1_ESM.zip › Figure 3D/P-GP.jpg]

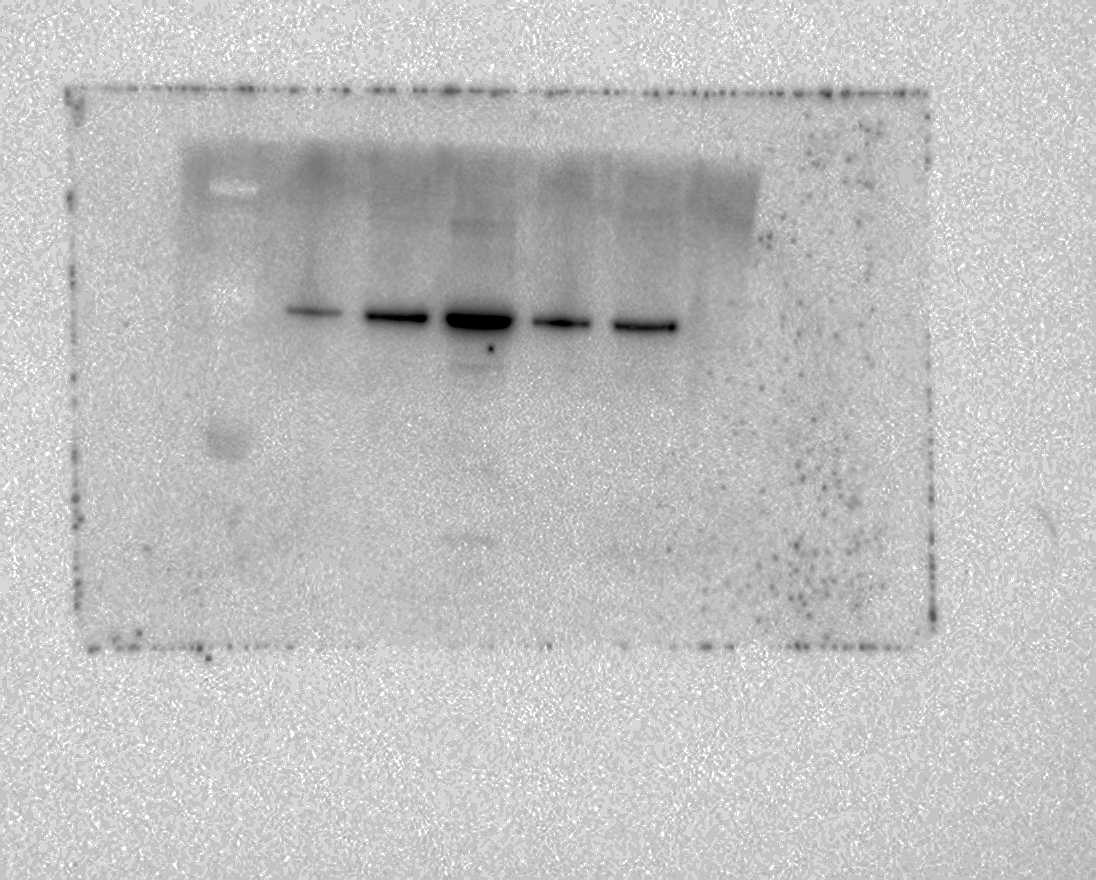

Supplement: Supplementary file 1 — Supplementary Material 1. [file 12894_2025_1994_MOESM1_ESM.zip › Figure 4F/BAX.jpg]

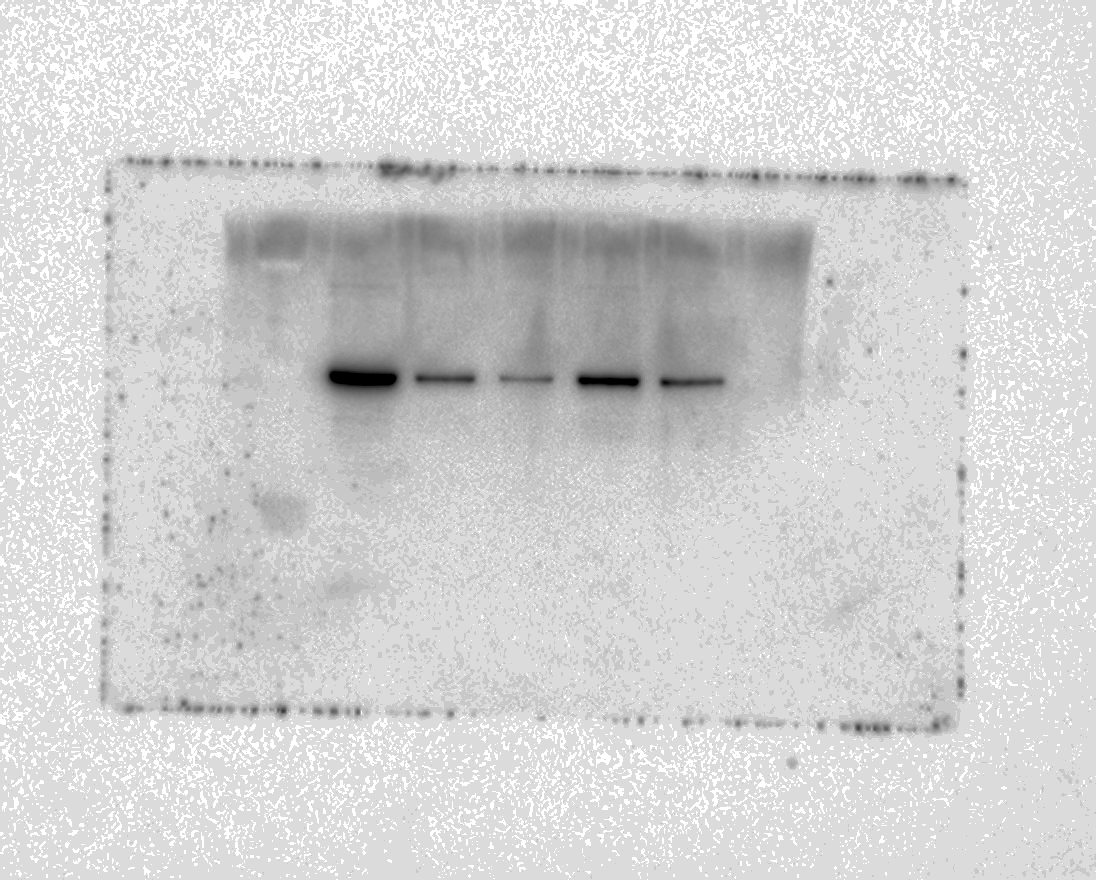

Supplement: Supplementary file 1 — Supplementary Material 1. [file 12894_2025_1994_MOESM1_ESM.zip › Figure 4F/BCL-2.jpg]

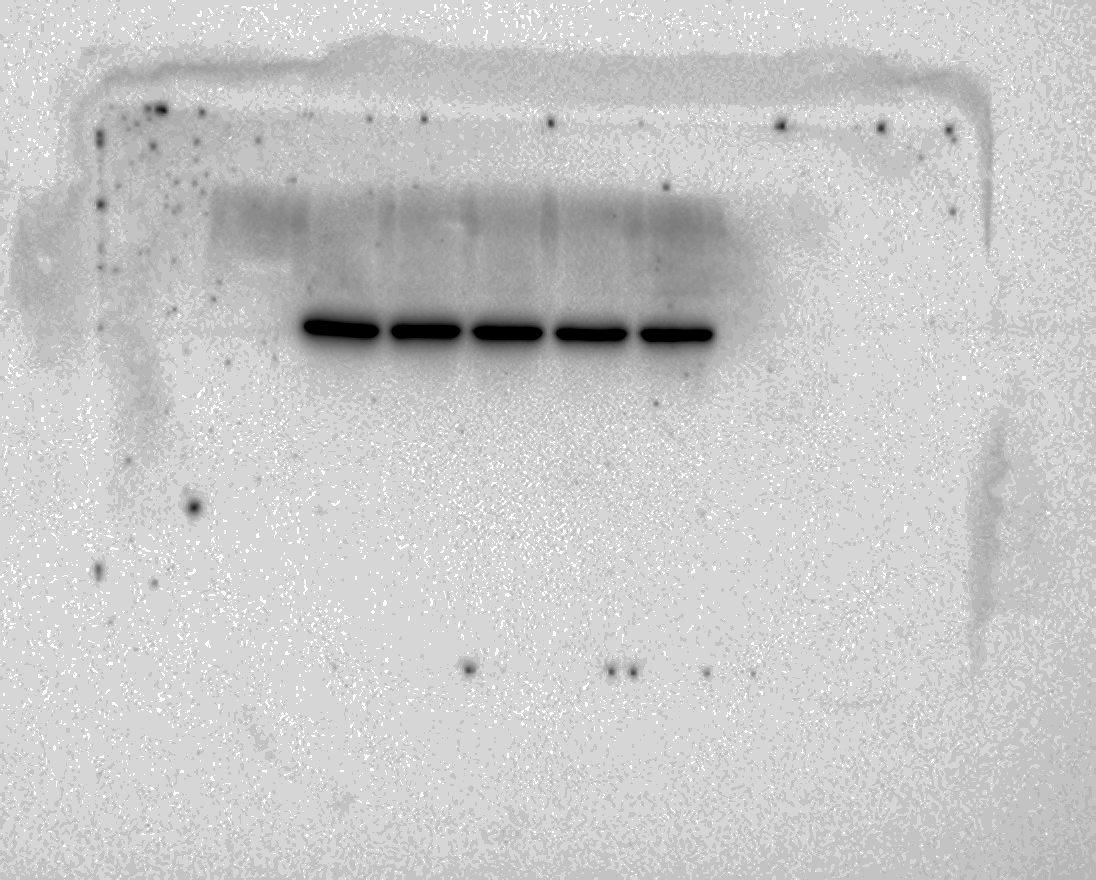

Supplement: Supplementary file 1 — Supplementary Material 1. [file 12894_2025_1994_MOESM1_ESM.zip › Figure 4F/GAPDH.jpg]

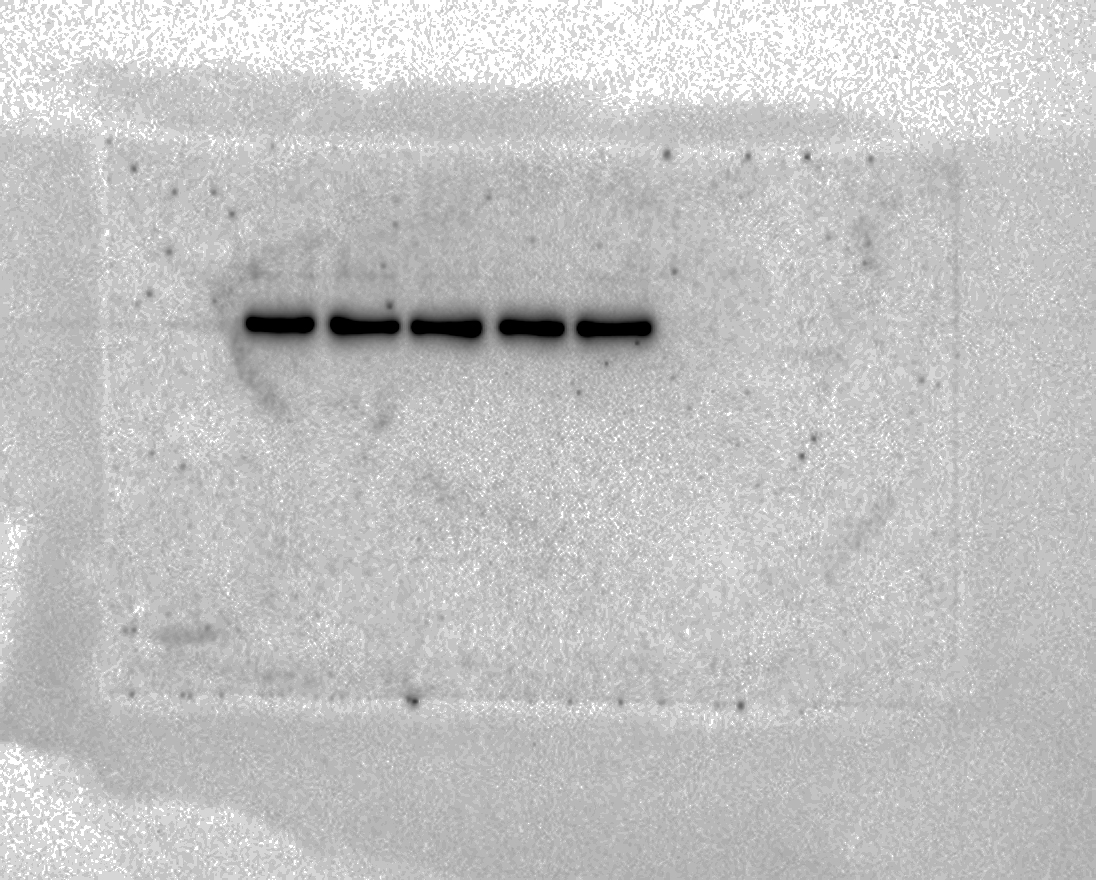

Supplement: Supplementary file 1 — Supplementary Material 1. [file 12894_2025_1994_MOESM1_ESM.zip › Figure 5B/GAPDH.jpg]

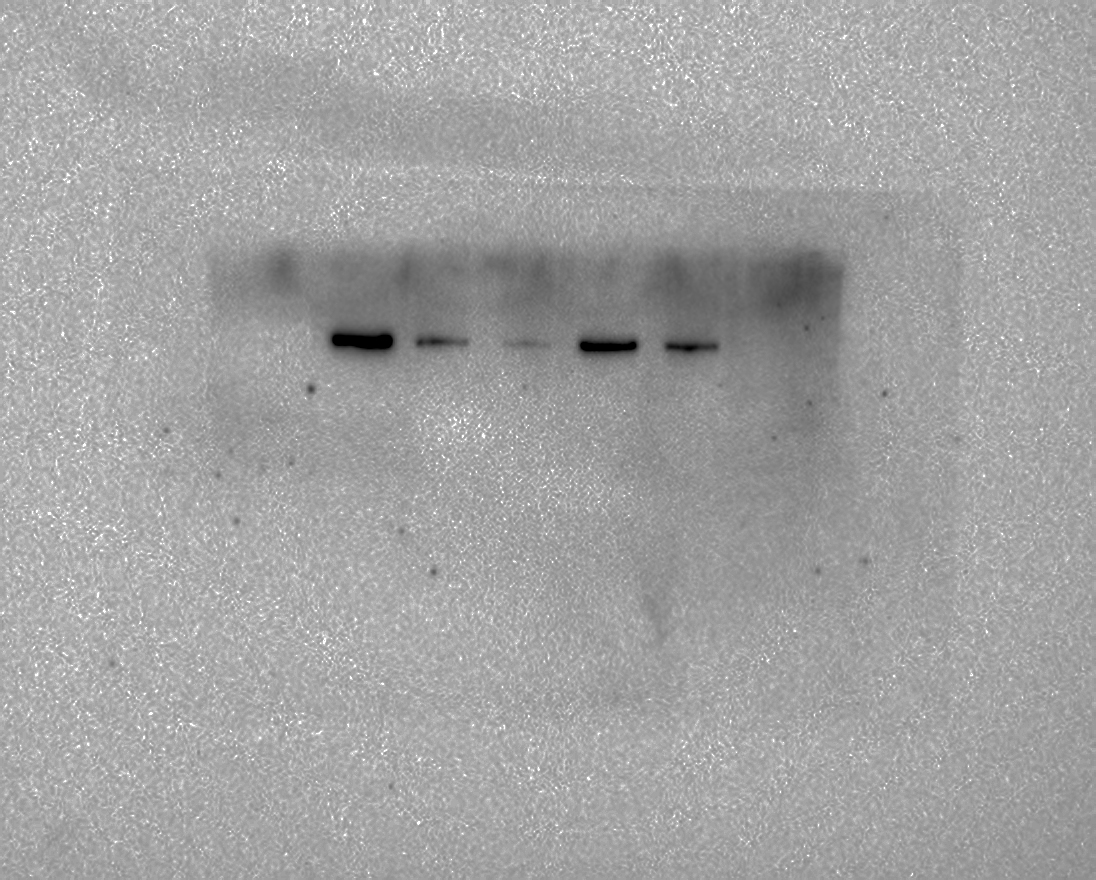

Supplement: Supplementary file 1 — Supplementary Material 1. [file 12894_2025_1994_MOESM1_ESM.zip › Figure 5B/GLUT1.jpg]

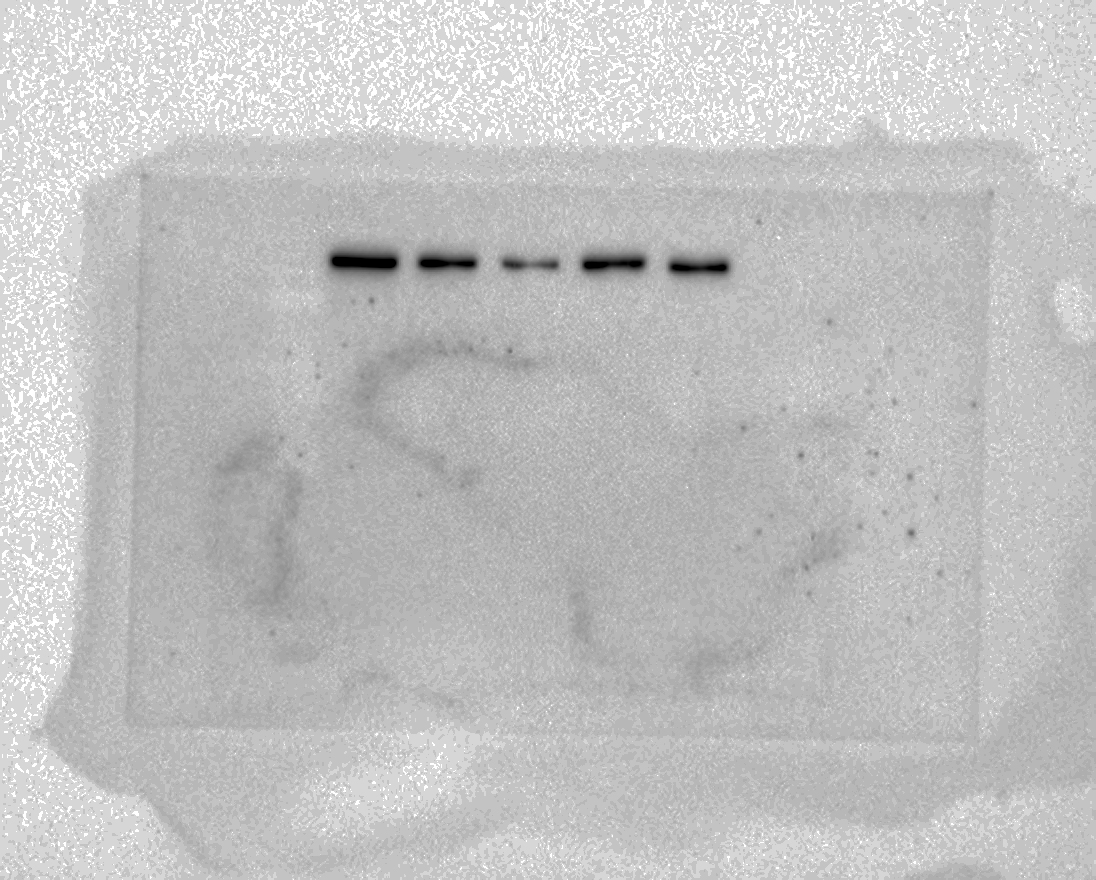

Supplement: Supplementary file 1 — Supplementary Material 1. [file 12894_2025_1994_MOESM1_ESM.zip › Figure 5B/HK2.jpg]

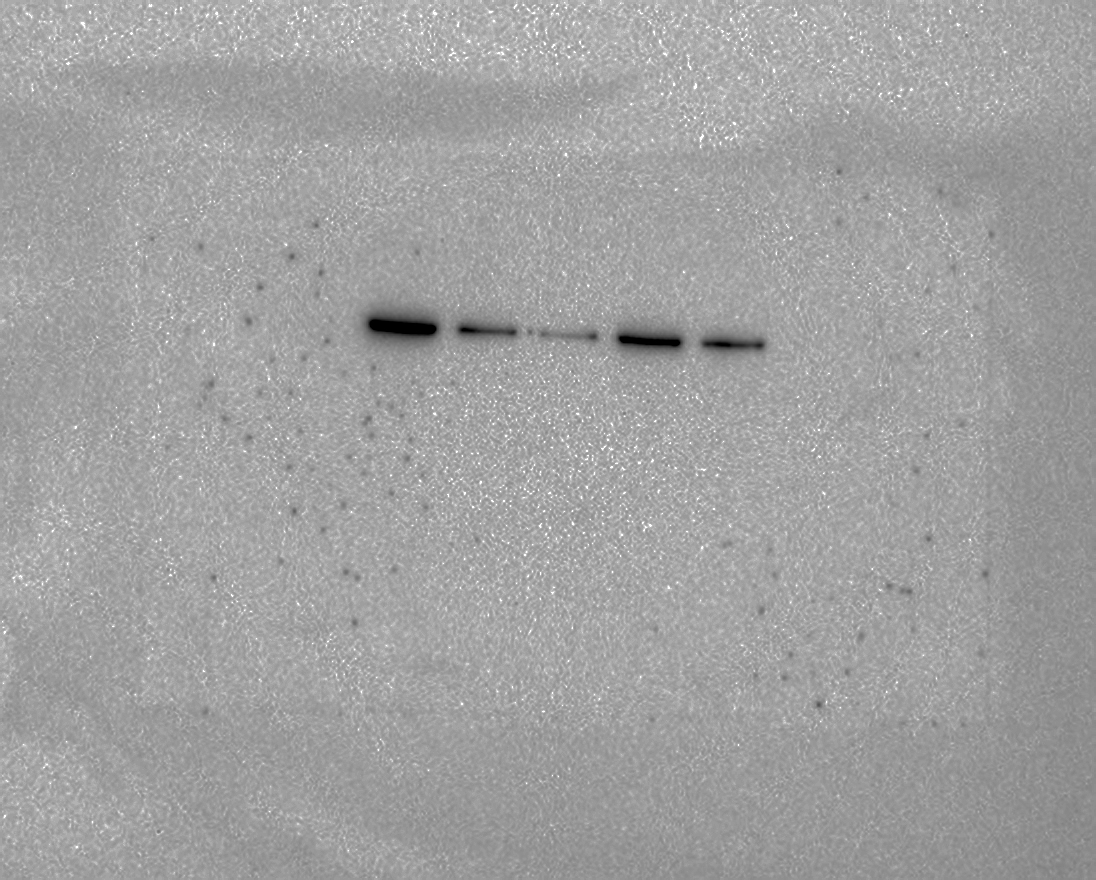

Supplement: Supplementary file 1 — Supplementary Material 1. [file 12894_2025_1994_MOESM1_ESM.zip › Figure 5B/LDHA.jpg]

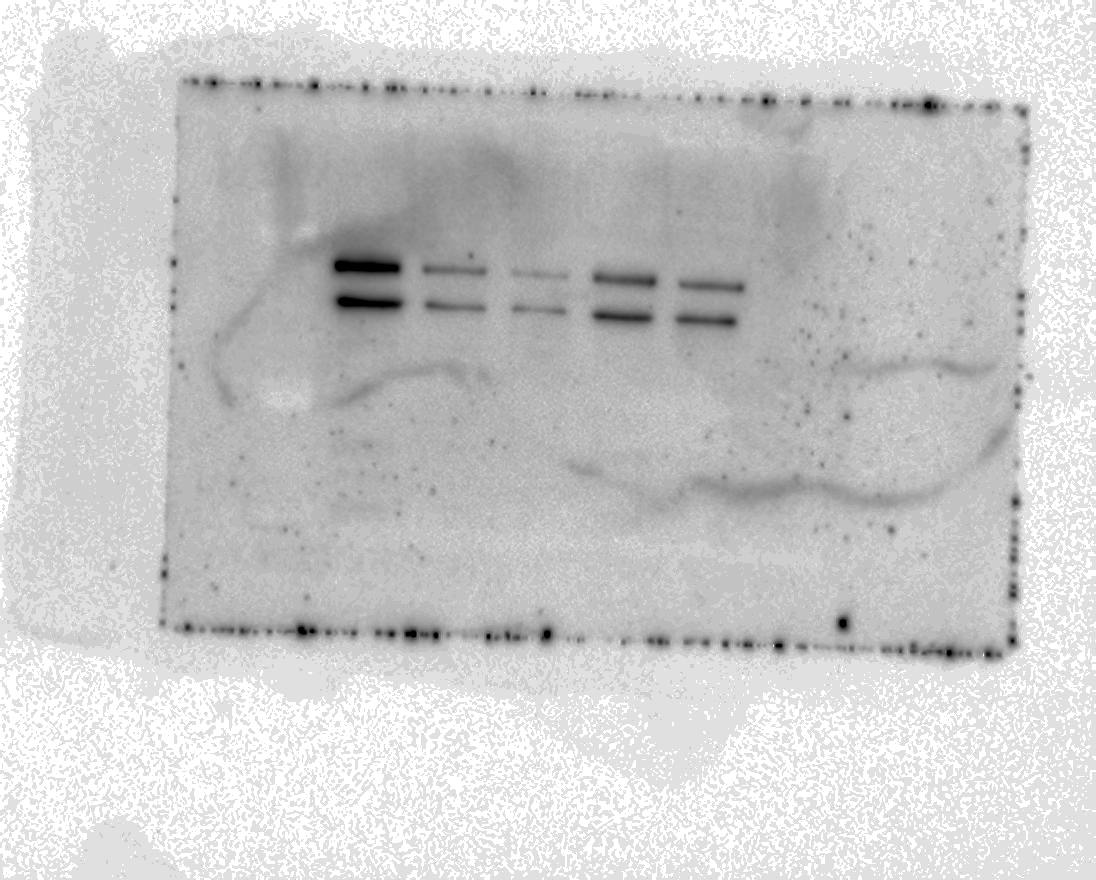

Supplement: Supplementary file 1 — Supplementary Material 1. [file 12894_2025_1994_MOESM1_ESM.zip › Figure 5C/C-MYC.jpg]

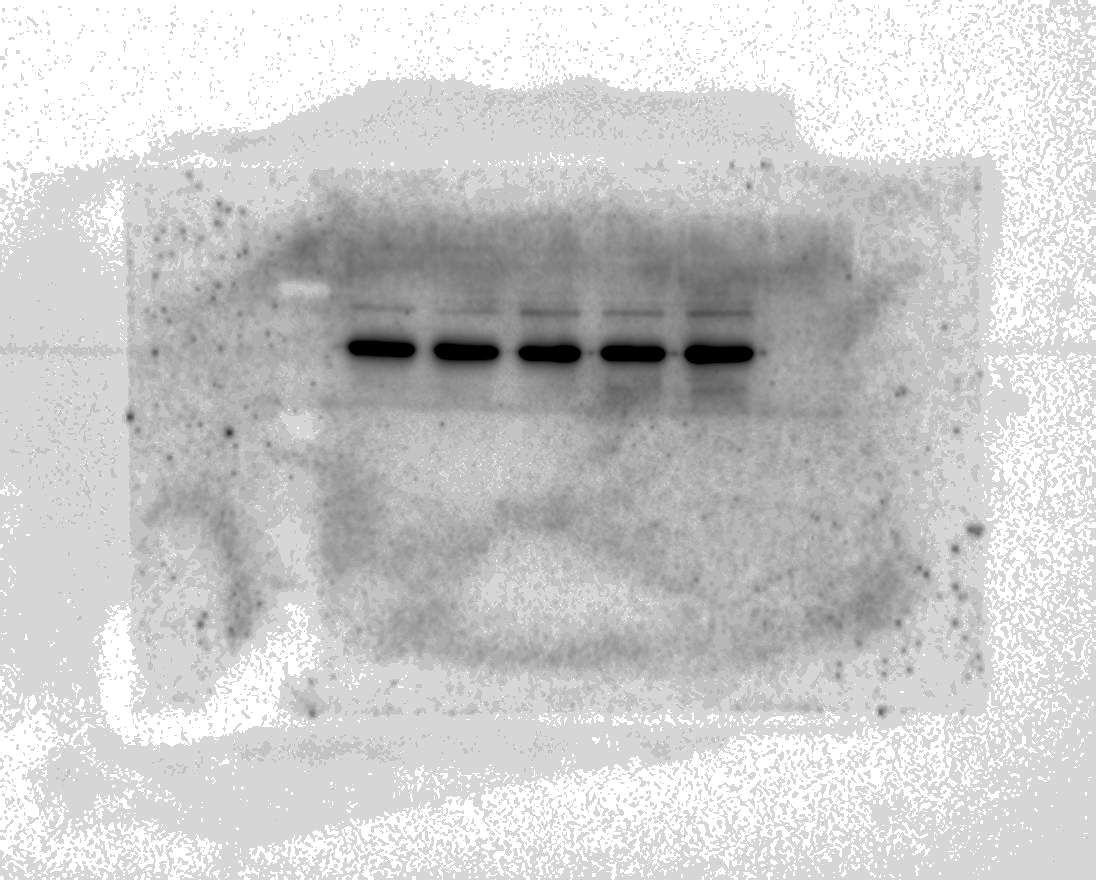

Supplement: Supplementary file 1 — Supplementary Material 1. [file 12894_2025_1994_MOESM1_ESM.zip › Figure 5C/GAPDH.jpg]

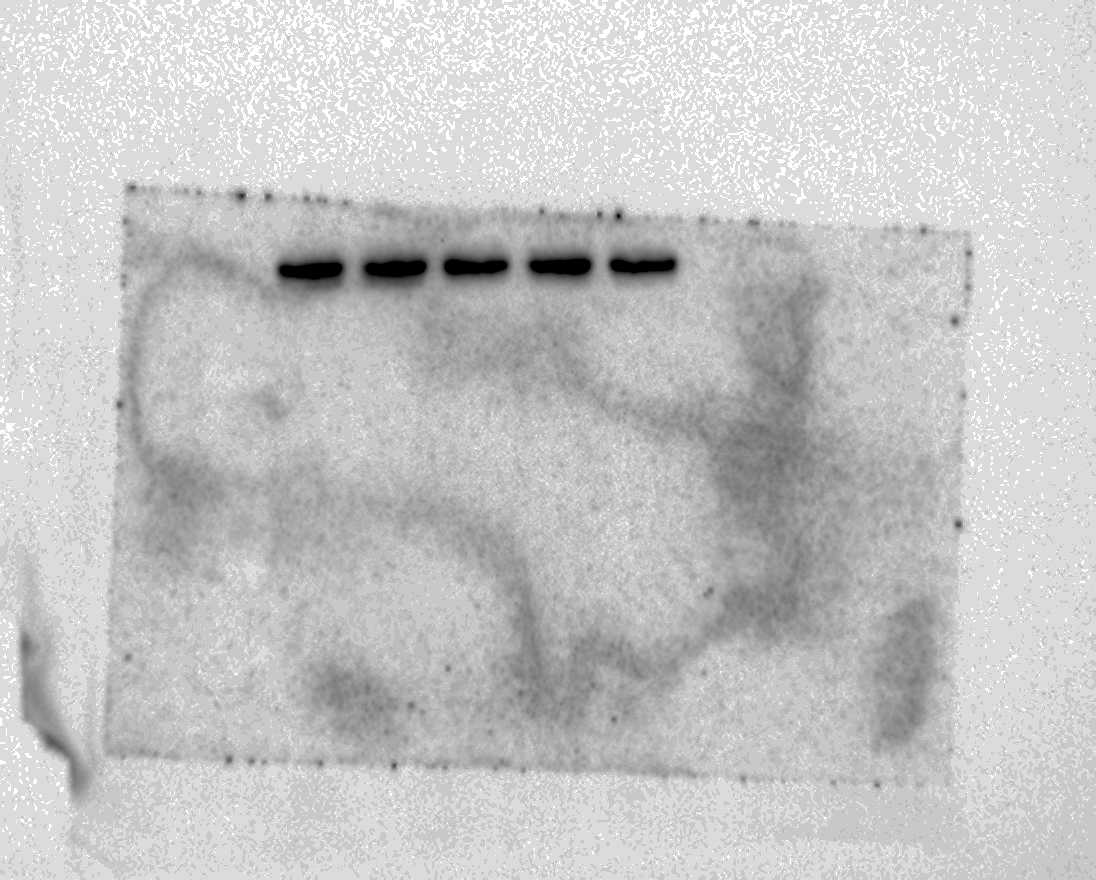

Supplement: Supplementary file 1 — Supplementary Material 1. [file 12894_2025_1994_MOESM1_ESM.zip › Figure 5C/mTOR.jpg]

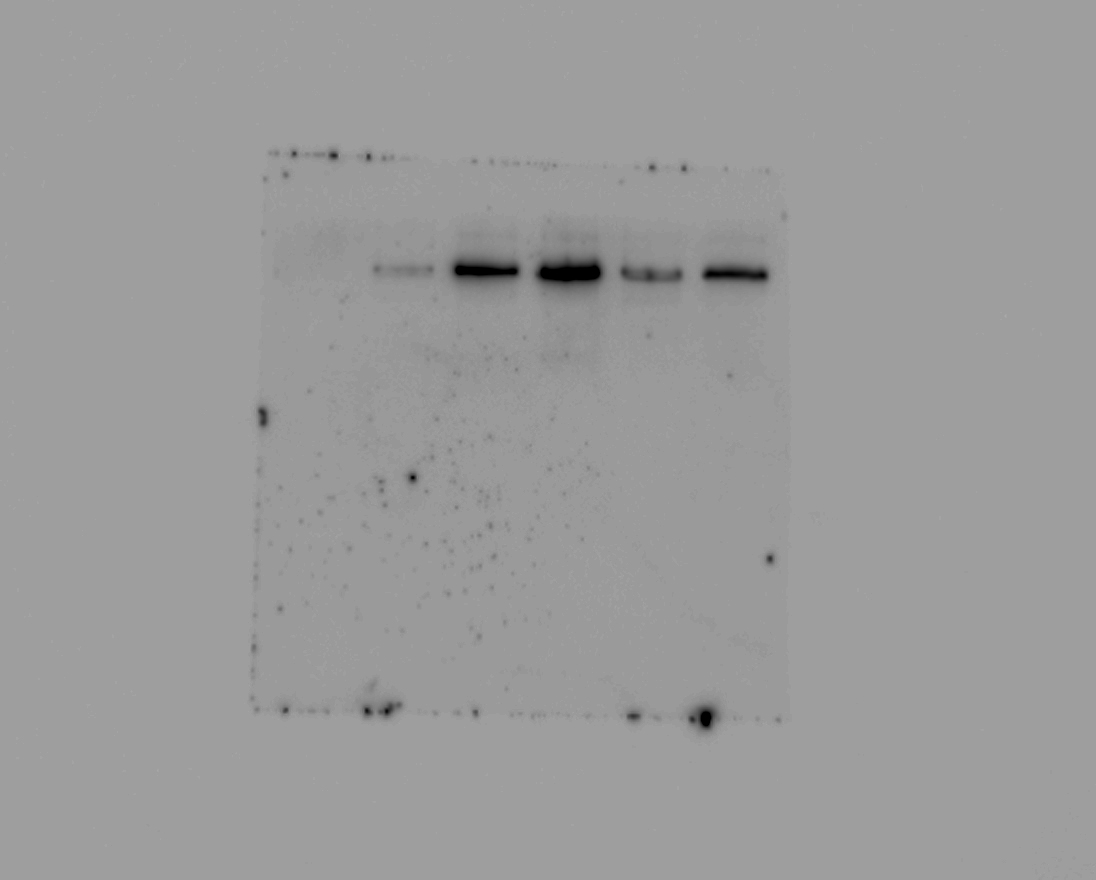

Supplement: Supplementary file 1 — Supplementary Material 1. [file 12894_2025_1994_MOESM1_ESM.zip › Figure 5C/P-MTOR.jpg]

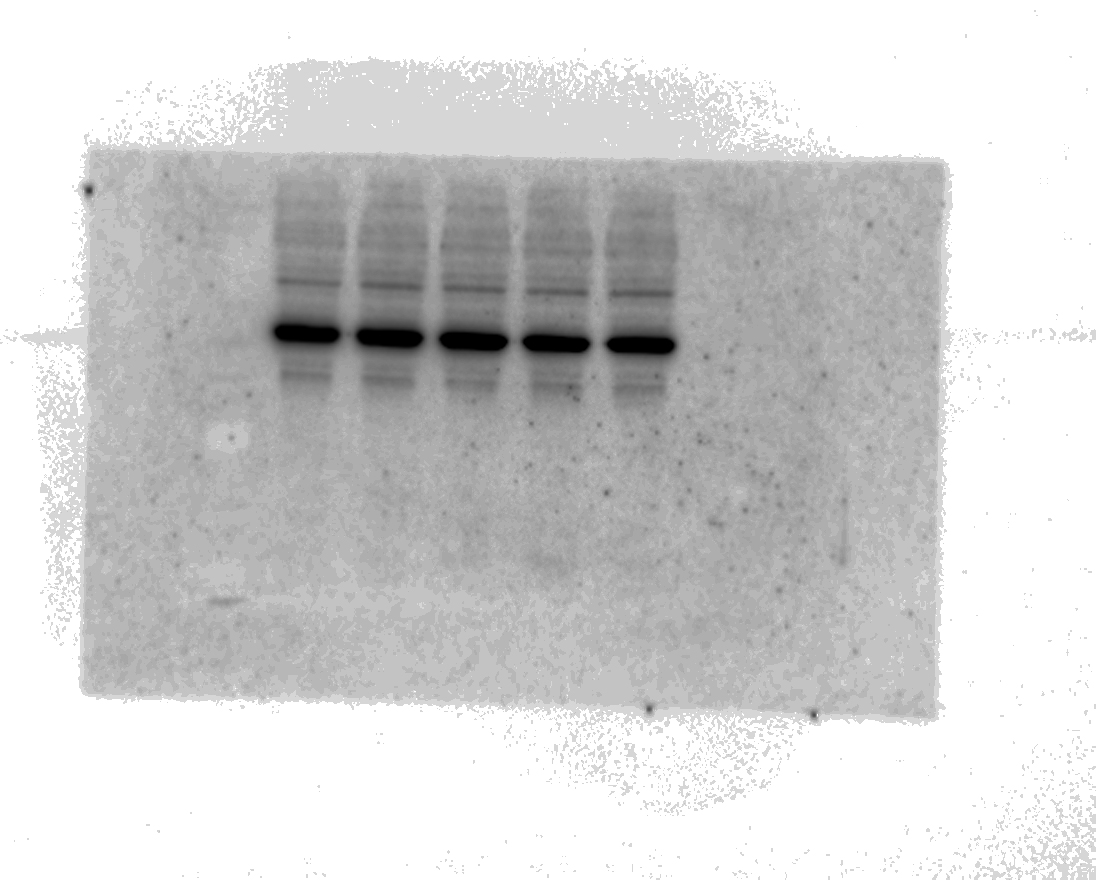

Supplement: Supplementary file 1 — Supplementary Material 1. [file 12894_2025_1994_MOESM1_ESM.zip › Figure 5D/GAPDH.jpg]

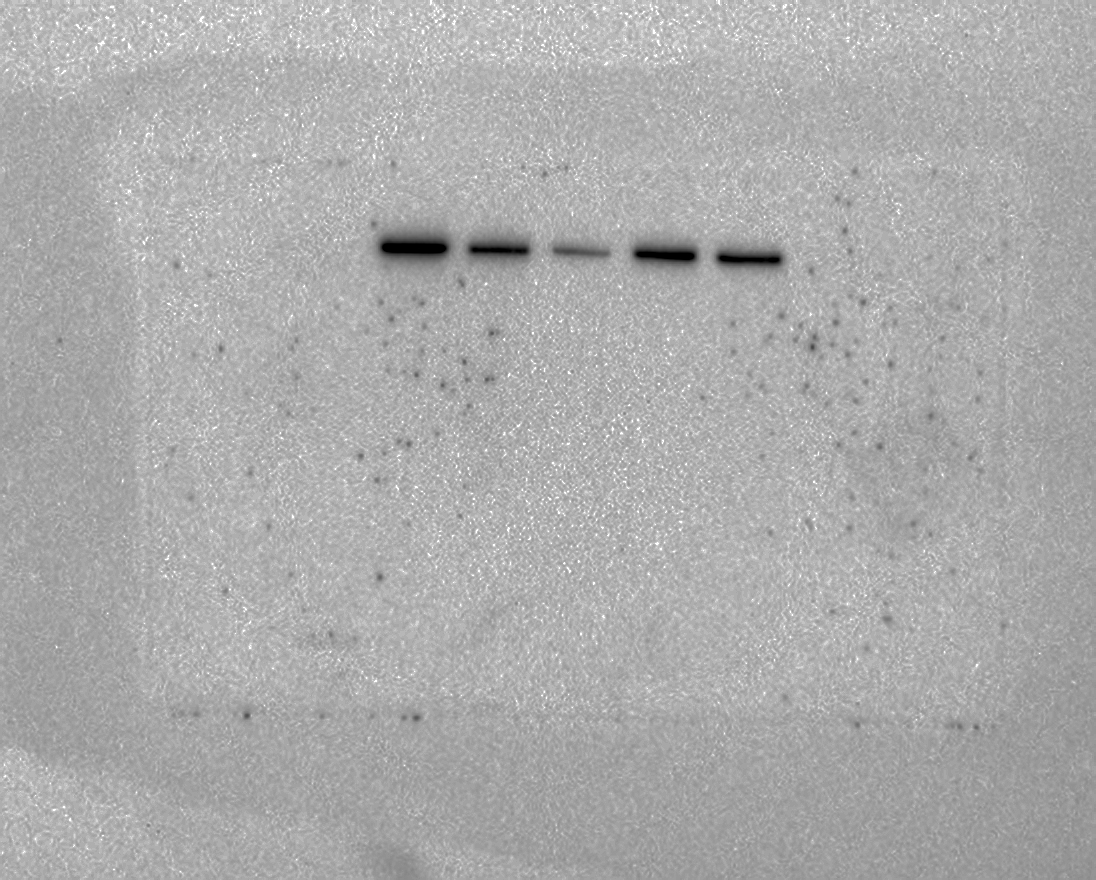

Supplement: Supplementary file 1 — Supplementary Material 1. [file 12894_2025_1994_MOESM1_ESM.zip › Figure 5D/MRP1.jpg]

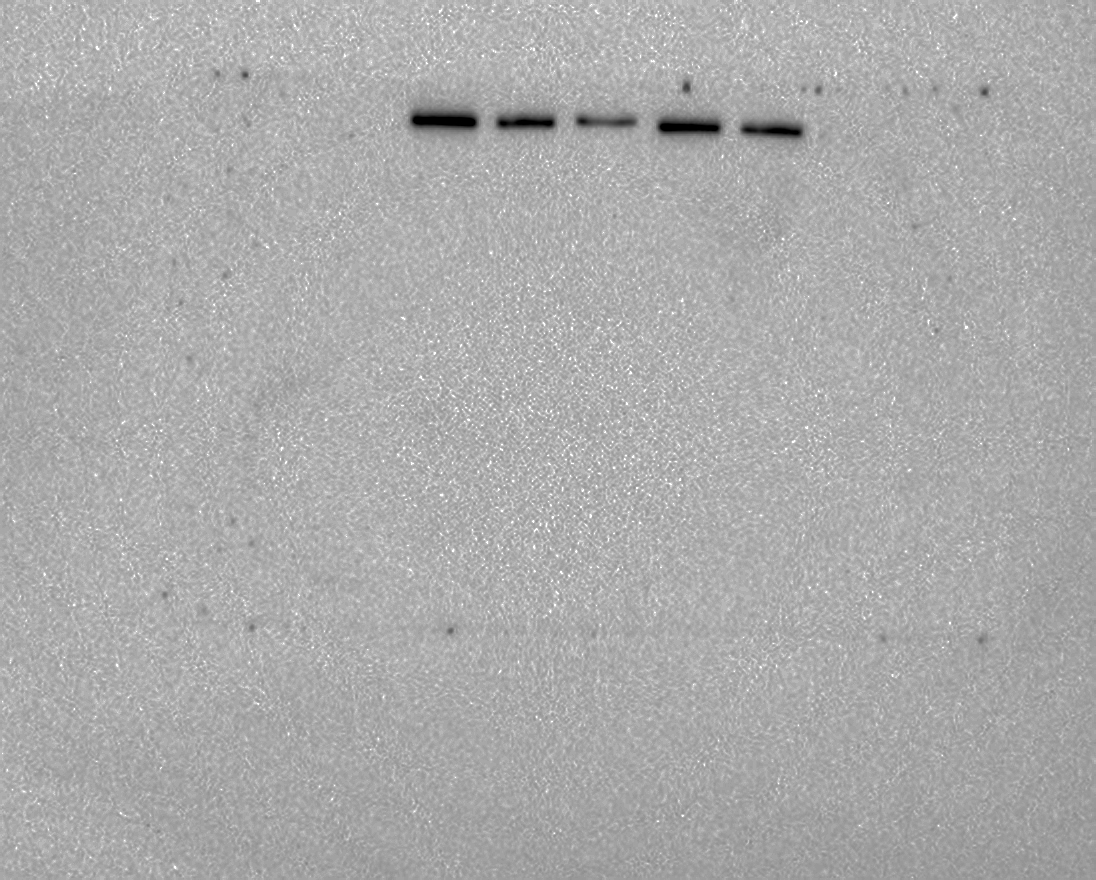

Supplement: Supplementary file 1 — Supplementary Material 1. [file 12894_2025_1994_MOESM1_ESM.zip › Figure 5D/P-GP.jpg]

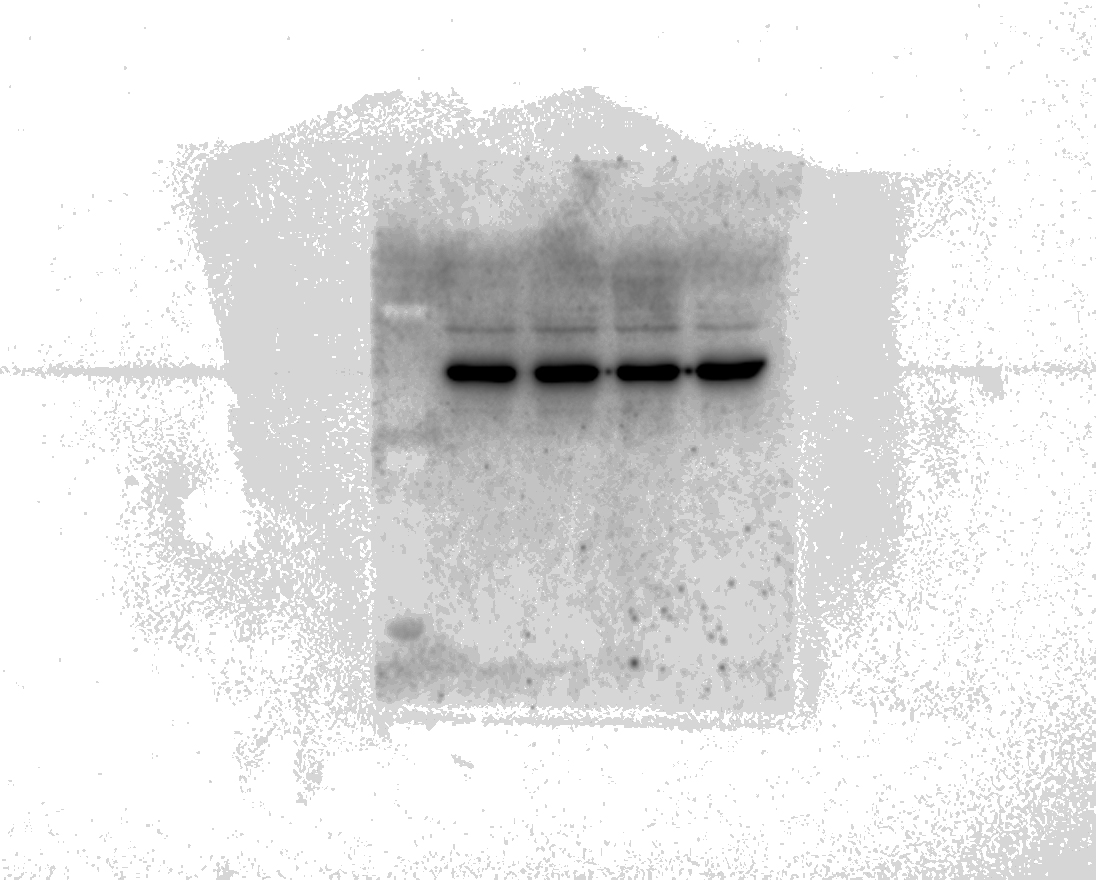

Supplement: Supplementary file 1 — Supplementary Material 1. [file 12894_2025_1994_MOESM1_ESM.zip › Figure 6C/GAPDH.jpg]

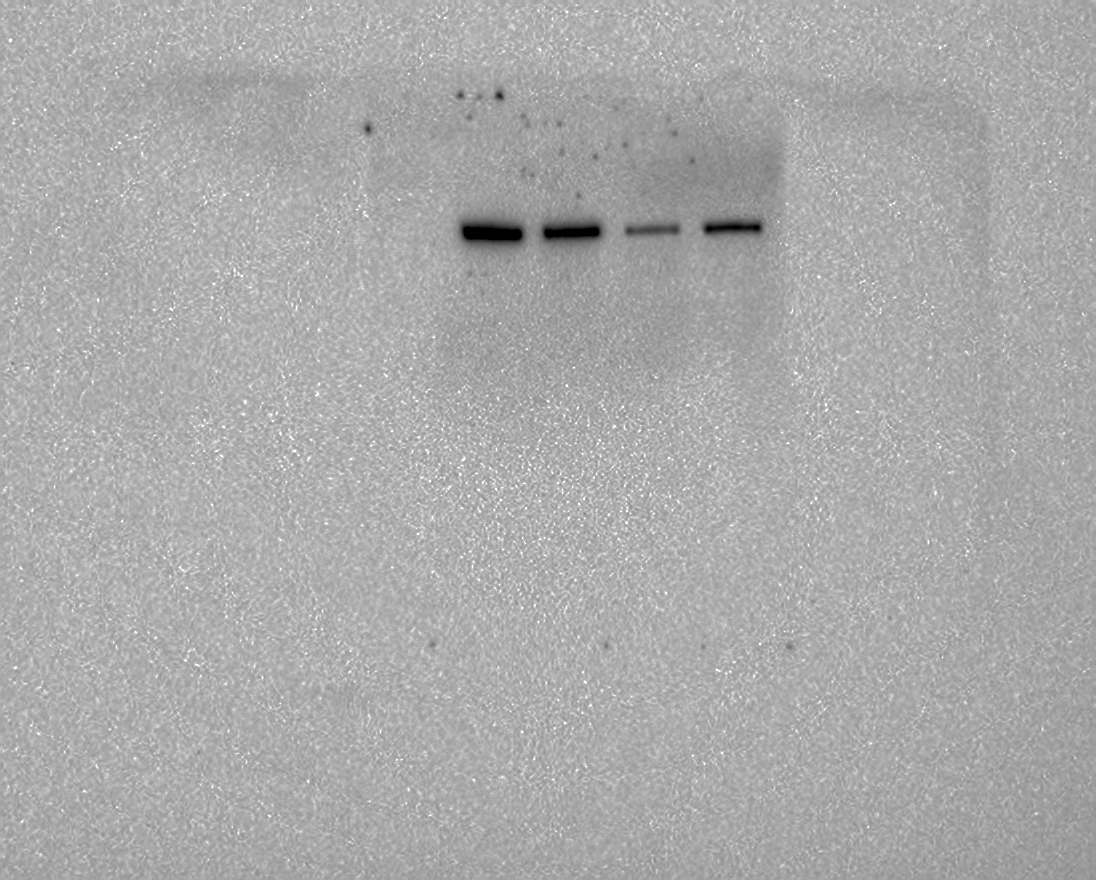

Supplement: Supplementary file 1 — Supplementary Material 1. [file 12894_2025_1994_MOESM1_ESM.zip › Figure 6C/GLUT1.jpg]

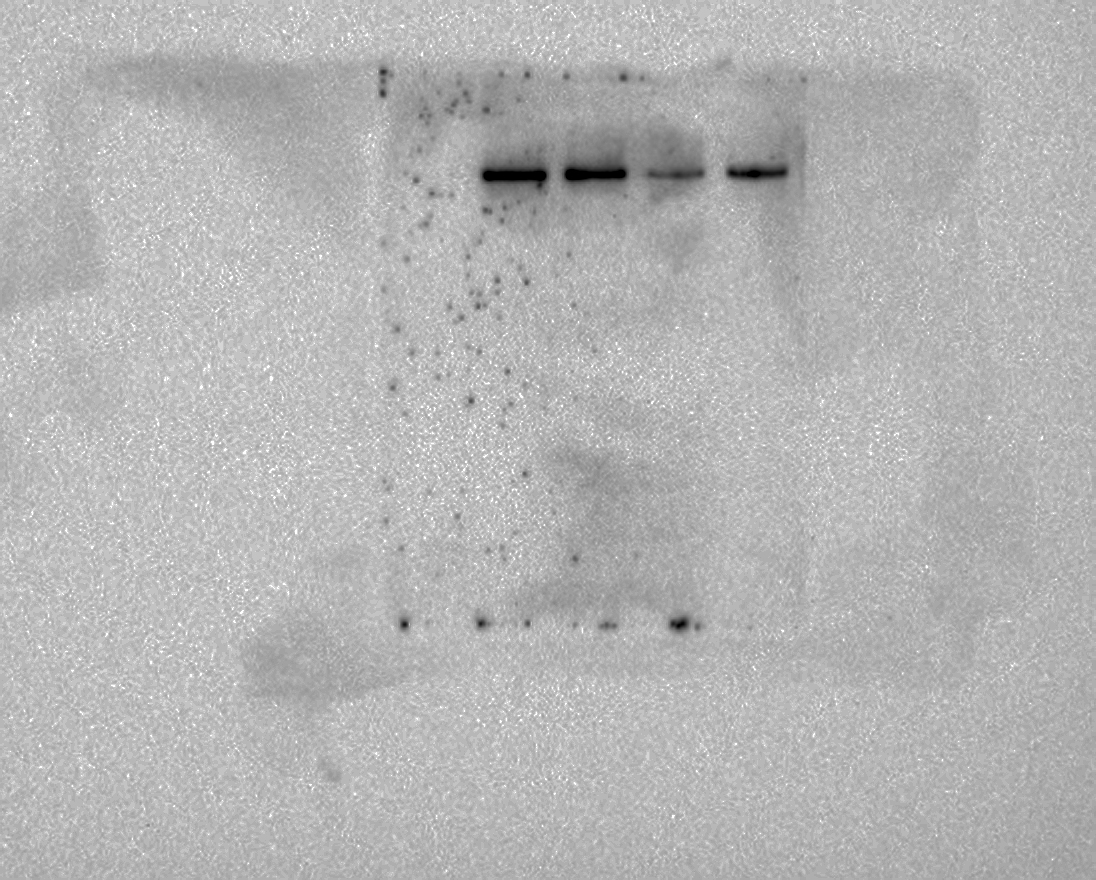

Supplement: Supplementary file 1 — Supplementary Material 1. [file 12894_2025_1994_MOESM1_ESM.zip › Figure 6C/HK2.jpg]

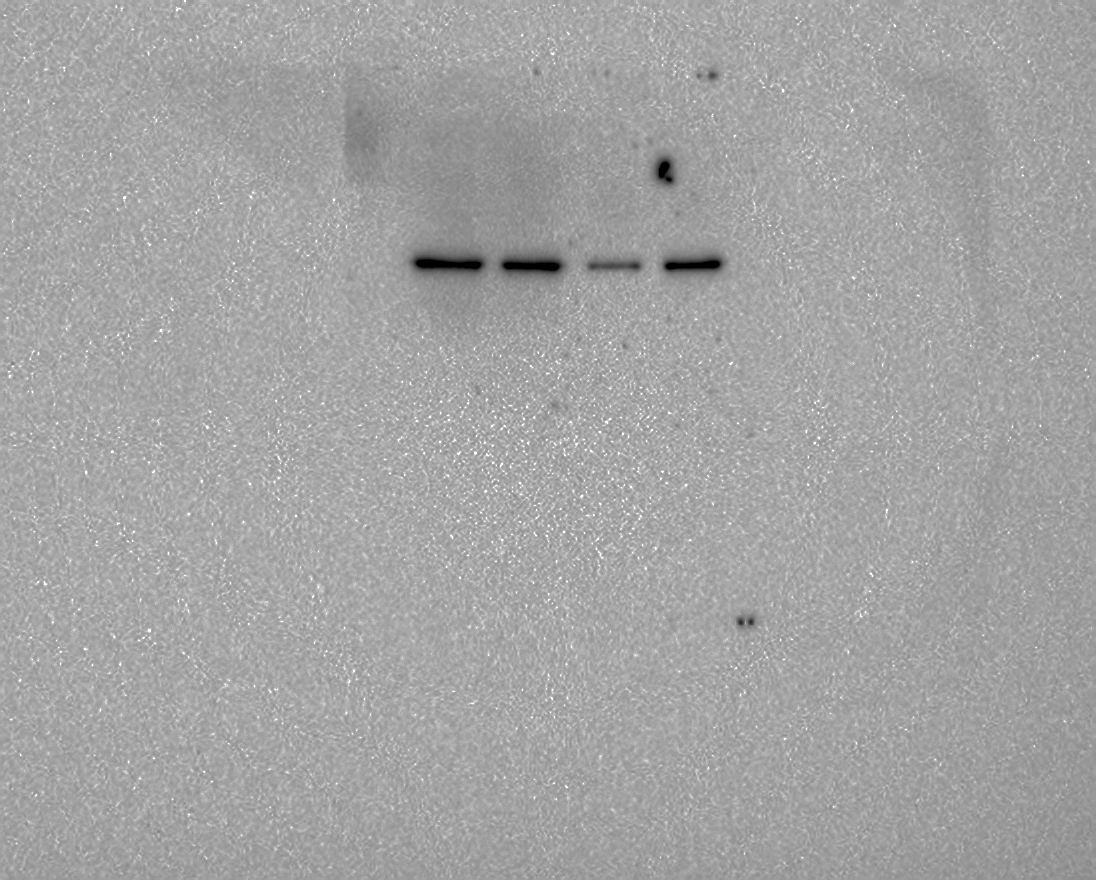

Supplement: Supplementary file 1 — Supplementary Material 1. [file 12894_2025_1994_MOESM1_ESM.zip › Figure 6C/LDHA.jpg]

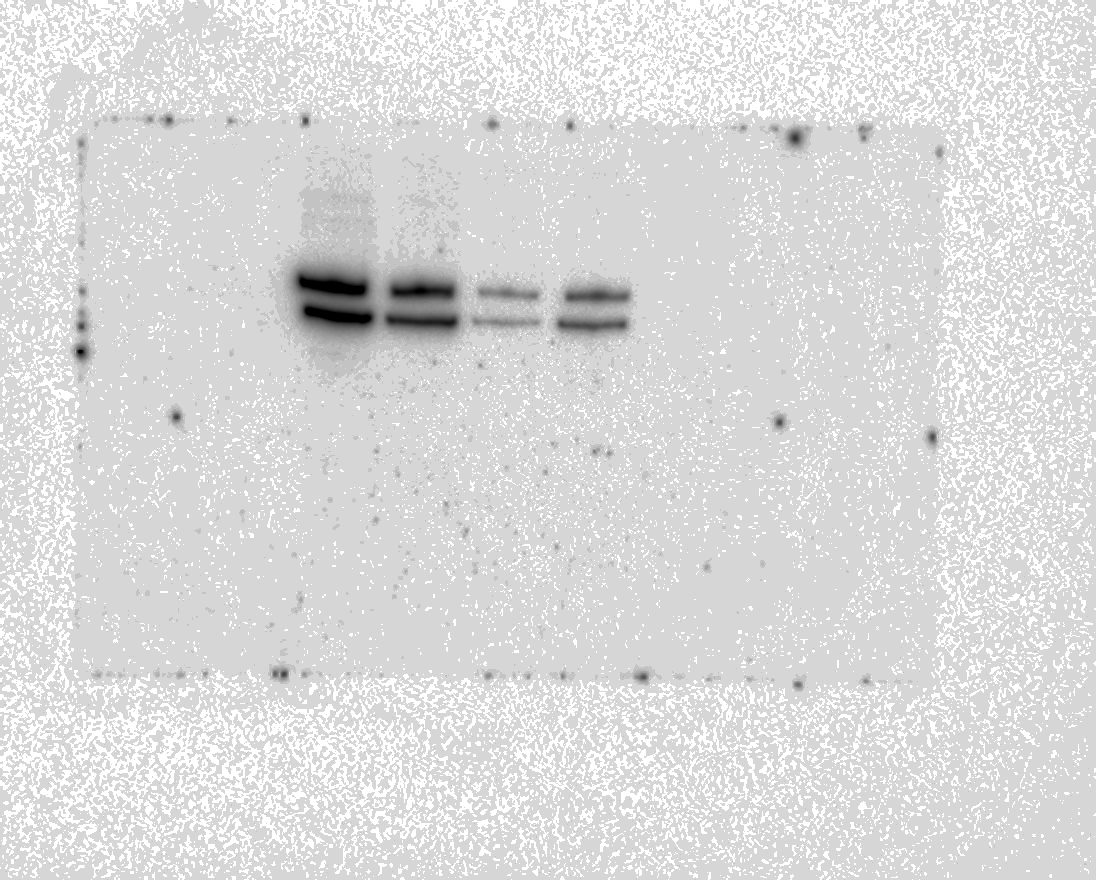

Supplement: Supplementary file 1 — Supplementary Material 1. [file 12894_2025_1994_MOESM1_ESM.zip › Figure 6D/C-MYC.jpg]

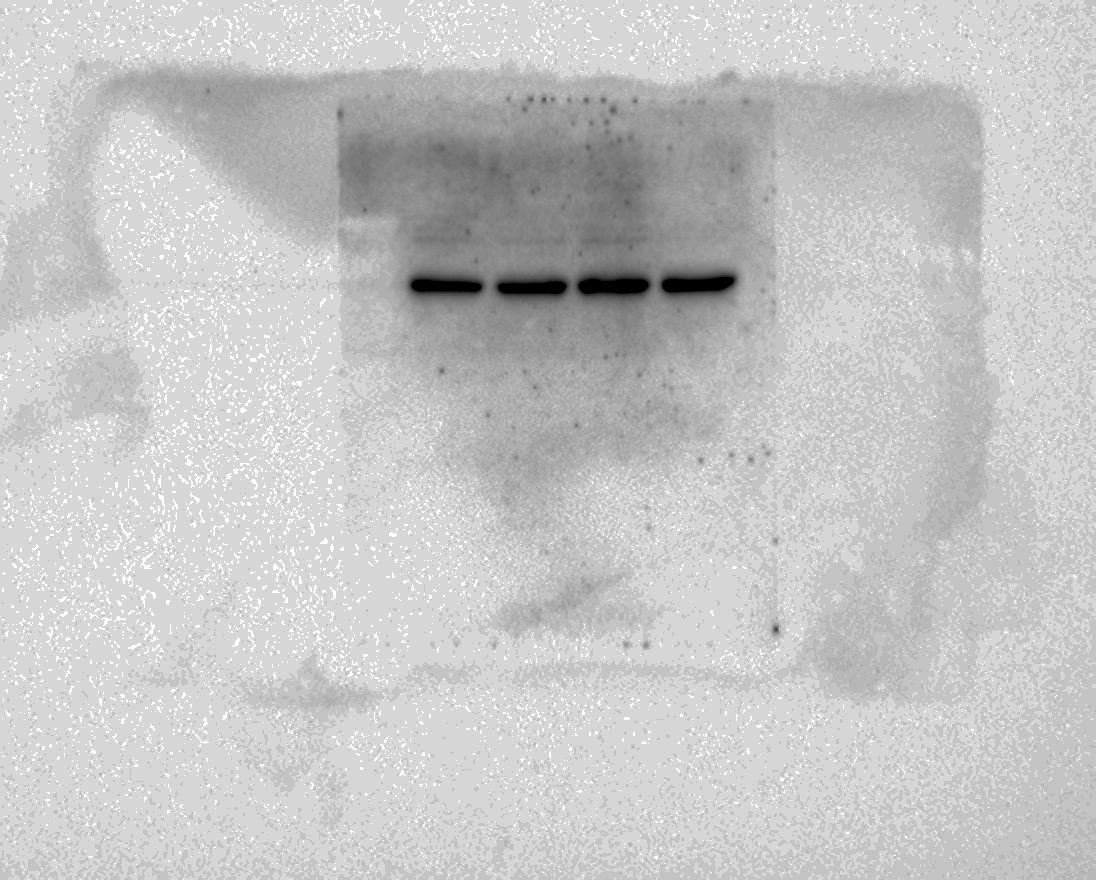

Supplement: Supplementary file 1 — Supplementary Material 1. [file 12894_2025_1994_MOESM1_ESM.zip › Figure 6D/GAPDH.jpg]

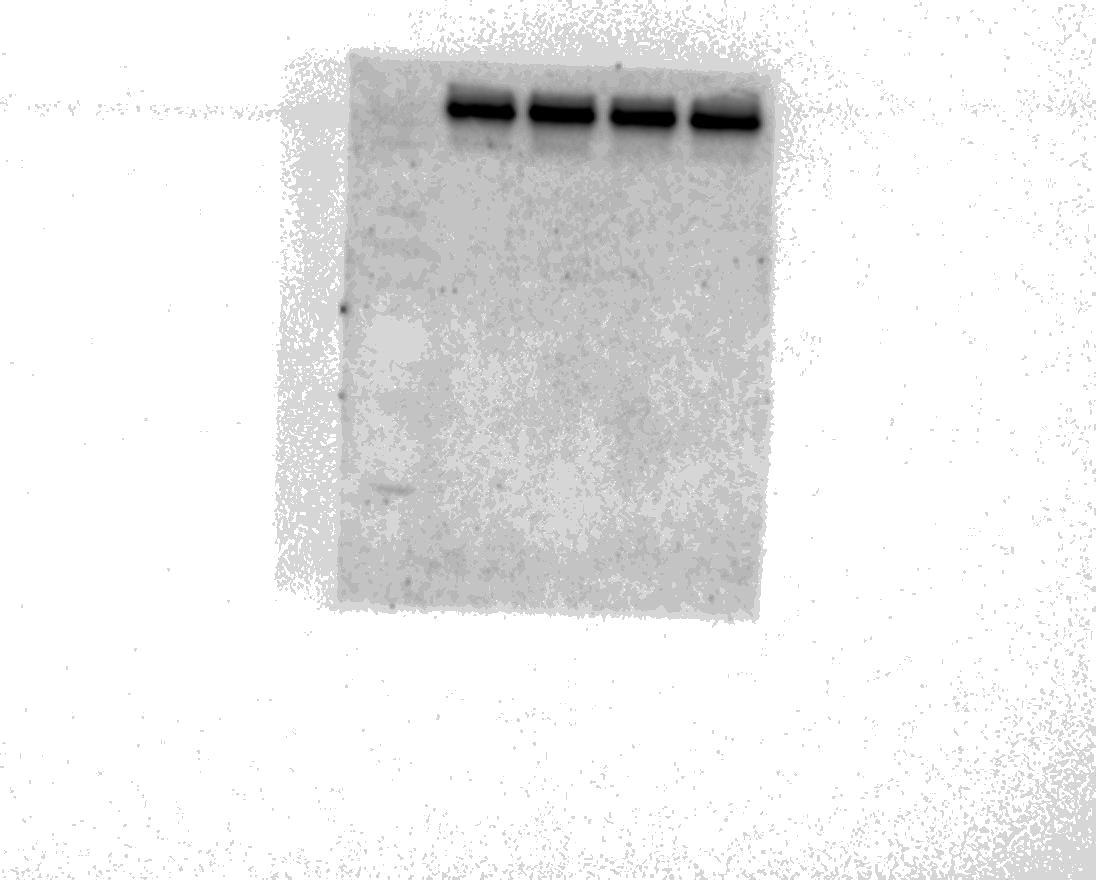

Supplement: Supplementary file 1 — Supplementary Material 1. [file 12894_2025_1994_MOESM1_ESM.zip › Figure 6D/Mtor.jpg]

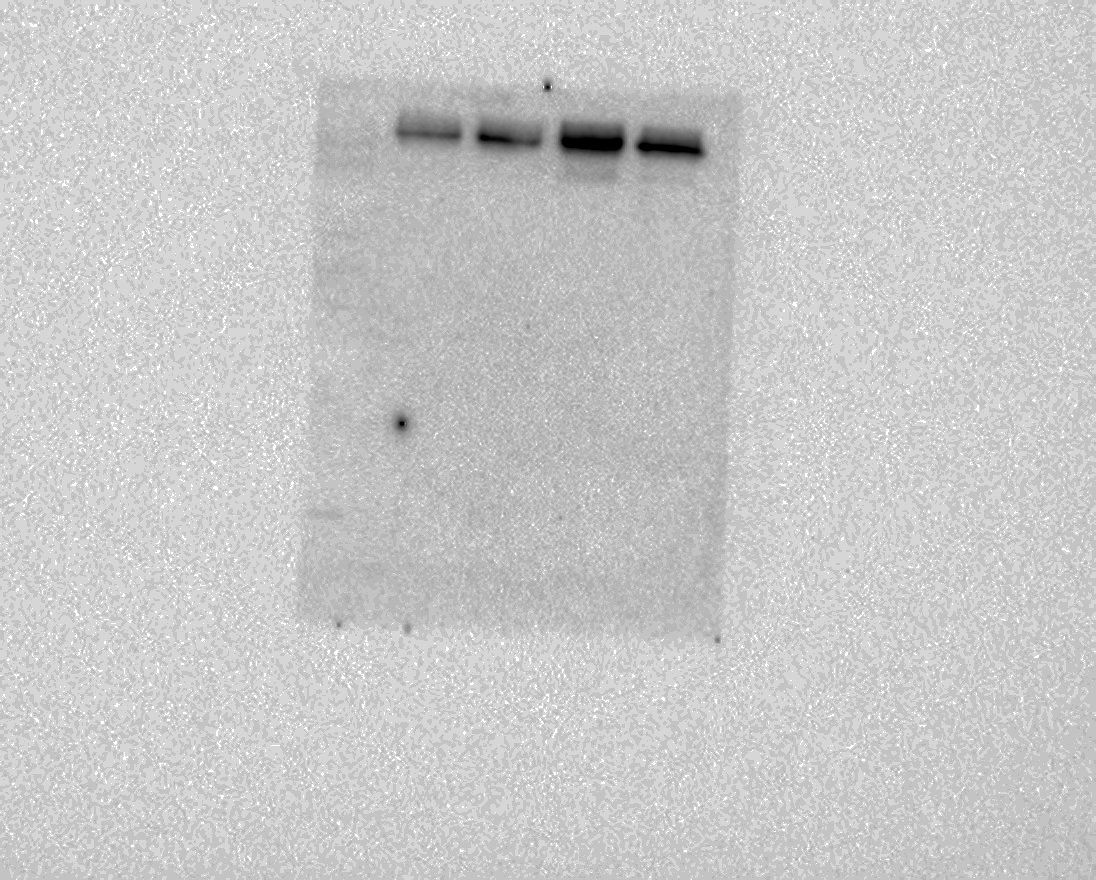

Supplement: Supplementary file 1 — Supplementary Material 1. [file 12894_2025_1994_MOESM1_ESM.zip › Figure 6D/P-Mtor.jpg]
