# Supplementary material for: Vitamin D promotes apoptosis and enhances cisplatin sensitivity in bladder cancer cells by inhibiting the Warburg effect through the AKT/mTOR pathway
Source: BMC Urol. 2025 Dec 15;26:11. doi: 10.1186/s12894-025-01994-2 (PMC12822197; doi:10.1186/s12894-025-01994-2)
Supplement: Supplementary file 2 — Supplementary Material 2. [file 12894_2025_1994_MOESM2_ESM.pdf]

Editing Certificate

This document certifies that the manuscript listed below has been edited to ensure language and grammar accuracy and is error free in these aspects. The edit was performed by professional editors at Editage, a brand of Cactus Communications. The author's core research ideas were not altered in any way during the editing process. The quality of the edit has been guaranteed, with the assumption that our suggested changes have been accepted and the text has not been further altered without the knowledge of our editors.

MANUSCRIPT TITLE

Vitamin D promotes apoptosis and enhances cisplatin sensitivity in bladder cancer cells by inhibiting the Warburg effect through the AKT/mTOR pathway

AUTHORS

Jian Zhou, Chaoyang Zhang, Xiao Wang, Ji Xing, Geng Cheng, Hao Chu

ISSUED ON

March 29, 2025

JOB CODE

NIUSA_148

THE EDITAGE Promise QUALITY GUARANTEE

Prabh Grewal
Senior Vice President - Editage

editage helping you get published

Since 2002, Editage has helped over 430,000 authors publish around 1.2 million research papers in scholarly journals across over 1000 disciplines through editorial, translation, transcription, and publication support services. Editage is a brand of Cactus Communications (cactusglobal.com), a science communication and technology company.

CACTUS.

GLOBAL :
+1(669) 272-1214 | request@editage.com

CHINA :
400-001-8237; 021-60209400 | fabiao@editage.cn
